# Cell-free synthetic biology for natural product biosynthesis and discovery

**DOI:** 10.1039/d4cs01198h

**Published:** 2025-03-19

**Authors:** Andrew J. Rice, Tien T. Sword, Kameshwari Chengan, Douglas A. Mitchell, Nigel J. Mouncey, Simon J. Moore, Constance B. Bailey

**Affiliations:** a Department of Biochemistry, School of Medicine – Basic Sciences, Vanderbilt University Medical Research Building-IV Nashville Tennessee 37232 USA; b Department of Chemistry, University of Tennessee-Knoxville Knoxville TN USA; c School of Biosciences, University of Kent Canterbury CT2 7NZ UK; d Department of Chemistry, Vanderbilt University, Medical Research Building-IV, Nashville Tennessee 37232 USA; e DOE Joint Genome Institute, Lawrence Berkeley National Laboratory Berkeley California 94720 USA; f Department of Life Sciences, Imperial College London London SW7 2AZ UK simon.moore@imperial.ac.uk; g School of Chemistry, University of Sydney Camperdown NSW 2001 Australia constance.bailey@sydney.edu.au

## Abstract

Natural products have applications as biopharmaceuticals, agrochemicals, and other high-value chemicals. However, there are challenges in isolating natural products from their native producers (*e.g.* bacteria, fungi, plants). In many cases, synthetic chemistry or heterologous expression must be used to access these important molecules. The biosynthetic machinery to generate these compounds is found within biosynthetic gene clusters, primarily consisting of the enzymes that biosynthesise a range of natural product classes (including, but not limited to ribosomal and nonribosomal peptides, polyketides, and terpenoids). Cell-free synthetic biology has emerged in recent years as a bottom-up technology applied towards both prototyping pathways and producing molecules. Recently, it has been applied to natural products, both to characterise biosynthetic pathways and produce new metabolites. This review discusses the core biochemistry of cell-free synthetic biology applied to metabolite production and critiques its advantages and disadvantages compared to whole cell and/or chemical production routes. Specifically, we review the advances in cell-free biosynthesis of ribosomal peptides, analyse the rapid prototyping of natural product biosynthetic enzymes and pathways, highlight advances in novel antimicrobial discovery, and discuss the rising use of cell-free technologies in industrial biotechnology and synthetic biology.

## Abstract 2

Cell free synthetic biology enables a broad range of approaches relevant to the generation, biosynthetic characterisation, bioengineering, activity profiling, and production of complex natural products.

## Graphical abstract

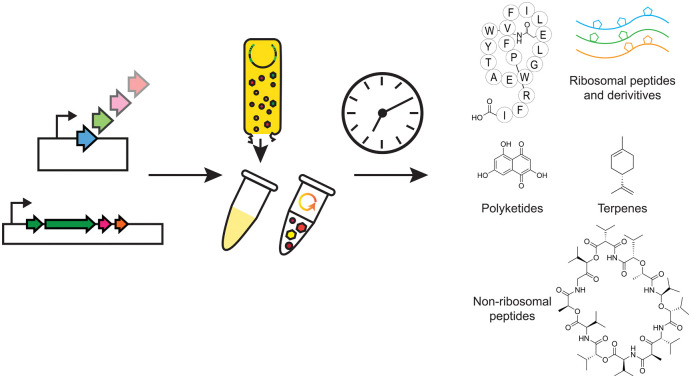

## 1. Introduction

Over the last 20 years, there has been a resurgence in using cell-free technologies – broadly referred to as cell-free synthetic biology – for a range of applications including the production of commodity and specialty chemicals, proteins, therapeutics, prototyping of gene expression, and diagnostics.^1,2^ However, only more recently have cell-free technologies been applied to complex and commercially relevant natural products. Historically, natural product biosynthesis has been investigated in an interdisciplinary fashion that draws upon synthetic chemistry, enzymology, genome mining, and cellular microbiology. As our ability to tackle complexity increases in cell-free synthetic biology, its applicability to natural product biosynthesis expands. This review summarises efforts to date and the strengths and limitations in applying cell-free transcription/translation systems to elucidating natural product biosynthesis and generating engineered secondary metabolites. This includes bioactivity-guided drug discovery, antimicrobial resistance (AMR) studies, and the growing role of cell-free innovation in industrial applications.^2^ Natural products have widespread applications and their chemical scaffolds are found in about one-third of U.S. Food and Drug Administration (FDA)-approved new molecular entities.^3^ They are an immensely important source of therapeutics, including but not limited to antimicrobial, anti-tumour, and anti-parasitic compounds. Many natural products have complex molecular architectures, are often greater than >500 Da, and thus do not always meet Lipinski's rule of five for drug-like chemical properties.^4^ In the past, the pharmaceutical industry discovered most of the antibiotic chemical scaffolds through Selman Waksman's approach of using high-throughput screening of extracts from a range of natural sources including environmental bacteria, fungi, and plants.^5^ In particular, the *Streptomyces* genus was a dominant source of antibiotics – *e.g.*, streptomycin, kanamycin, griseomycin.^6^ This period, known as the “Golden Age of Antibiotics,” has long since declined due to the rediscovery of known compounds, and the shift of the pharmaceutical industry to synthetic chemistry.

Together with traditional chemistry approaches, the emergence of both DNA recombinant technologies and enzymology studies aided several chemistry and chemical biology groups to decipher the biosynthesis of some of the most prominent natural products, successfully correlating genes to molecules. The release of the model *Streptomyces coelicolor* A3(2) genome sequence in the early 2000s provided a significant advance.^7^ Its genome contained clusters of genes spread throughout its linear chromosome that were linked to the biosynthesis of known natural products as biosynthetic gene clusters (BGCs). However, *S. coelicolor* A3(2) possessed 27 BGCs, which was a much higher number than the known natural products identified under standard laboratory culture conditions.^8^ With further research, 17 out of these 27 BGCs now have been assigned to known metabolites.^8^ As genome sequencing became more widely available, this pioneering work inspired a new era of using genomics to guide natural product discovery. Now, there are about 1.2 million bacterial genomes fully sequenced and approximately half a million sequenced metagenomes.^9^ Combined with advances in freely available genome mining tools such as antiSMASH, BAGEL, RODEO, ARTS and many others,^10–15^ this rich pool of genomic data suggests that the true chemical diversity of natural products vastly exceeds the number of known natural products,^16–18^ which have mostly been discovered through traditional bioactivity-guided isolations from crude extract and fractionation methods.^19–21^ Many of these advances have been driven by improved capacity in high-throughput screening, heterologous expression and genome and pathway engineering^8,22–26^ across a range of organisms.^27^

## 2. Cell approaches to natural product discovery.

Microbes generate natural products in their environment to help them survive, often triggered in response to specific chemical or physical cues, or other stress factors leading to genomic changes and adaptive evolution triggering BGC gene expression. Those BGCs that cannot be correlated to known natural products are often called “silent” or “cryptic”.^28^ “Silent” BGCs are transcriptionally or translationally dormant under standard laboratory conditions. “cryptic” BGCs refer to lesser characterised BGCs, where knowledge of the regulation or biosynthetic genes is incomplete, missing or unknown (*i.e.*, abundance of hypothetical genes, domains of unknown function), or spread throughout the genome rather than clustered. In addition, some natural products and pathway intermediates are labile. Therefore, in general, isolated natural products tend to be highly abundant and chemically stable in metabolite extracts. Considerable efforts have been put forth to elicit BGC activation in natural hosts, such as the one-strain many compounds (OSMAC) approach. In OSMAC, microbial isolates are cultured under an array of conditions to activate different BGCs.^29^ Other screening efforts include small molecule libraries to elicit BGC activation^30,31^ and co-culturing to simulate environmental competition and collusion between microbes.^32–34^ Genetic manipulations have been performed to express BGCs, such as over expressing or deleting regulators, replacing promoters or using heterologous expression systems. However, in general, discovery of molecules appropriate as therapeutics from natural sources often requires further modification of molecular structure beyond those found in naturally occurring molecules. Frequently, there is a need to manipulate enzymes and biosynthetic pathways with precise control to alter the molecular structure of natural products and improve bioactive properties. This can be to improve target affinity, but also to improve drug properties. In many cases, natural products are not optimised for pharmacological interactions with human physiology as they evolved in a distinct environment within the producing organism. These molecules typically must be further altered for mammalian cellular penetrance, reduced cytochrome P450 metabolism, and other properties that affect pharmacokinetics and toxicity.^20,35^ Given the immense molecular complexity of many natural products, analogue generation presents a significant barrier to their development as therapeutics.^36^

## 3. Why cell-free synthetic biology?

Synthetic biology holds promise in generating new natural products by enabling activation of “silent” or “cryptic” BGCs or allowing precise control over molecular structures. However, there are challenges associated with cell-based synthetic biology. It is often necessary to screen multiple genetic designs through iterative and resource-intense experiments that can be challenging, especially if a less genetically tractable or slower growing heterologous host is used, or if the intermediate/product is toxic.

**Fig. 1:**
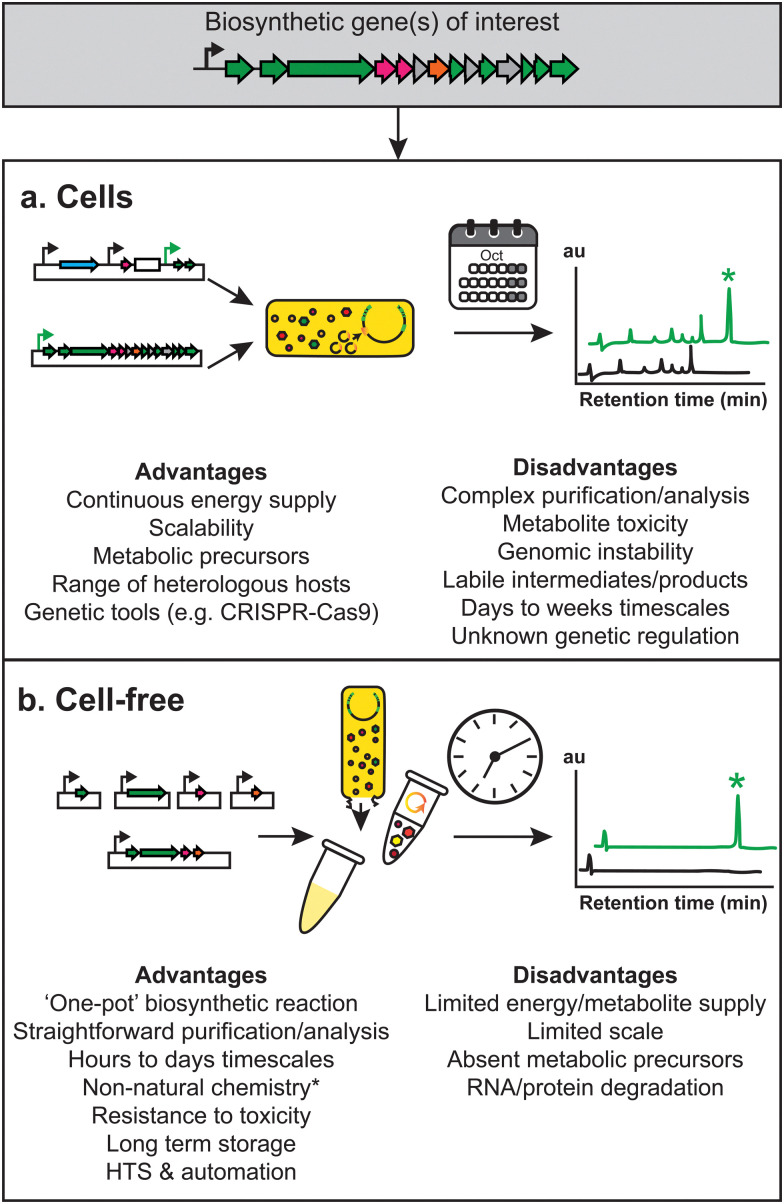
Overview of the advantages and disadvantages of (a) whole cell and (b) cell-free approaches to natural product biosynthesis and discovery.

Cell-free technologies have emerged as an alternative tool to accelerate the discovery and development of natural products and their derivatives. We focus here on describing the emerging uses of cell-free gene expression (CFE) for engineering natural product biosynthesis. By removing the cell wall, cell membrane and genomic DNA, cell-free extracts provide a quasi-chemical bioreactor platform, which can be modularly controlled to both make^1,37,38^ and detect^39–42^ RNA, peptides, proteins, and small molecules. Cell-free enzymes and ribozymes (*i.e.*, ribosomes) provide a powerful catalytic unit with the dexterity to study biological chemistry, and flexibility to work at different levels of scale from microfluidics^43,44^ through to 100 L reactions,^45–47^ and with evidence of linearity and low variability across these scales.^45^ Depending on the analysis type, CFE experiments can take a few minutes to hours,^48–50^ whereas depending on the context, a cell-based approach can take several days to weeks, potentially even longer if one performs genetic modification. Thus, CFE enables rapid cycling between experimental design and analysis^51^ of mRNA and protein synthesis.^44,52,53^ The starting concentration of the substrates and proteins can be determined,^52^ or controlled (*i.e.*, addition of purified proteins and chemicals), aiding standardisation and predictive modelling.^51,54^ CFE reactions can be extended by replenishment of the reaction substrates and removal of metabolic end-products (*e.g.*, lactate, acetate), through dialysis or microfluidics devices, extending steady-state protein synthesis for up to 30 hours.^44^ To increase the speed, or directly engineer the DNA templates, CFE reactions can work with linear DNA products.^55^ In addition, since cell-free extracts are non-living, they are safer to explore with low biocontainment facilities and expertise. CFE can also be freeze-dried to facilitate transport at room temperature, and later use upon rehydration.^39,56^ These collective advantages make CFE increasingly attractive to chemists, chemical engineers, and synthetic biologists. However, there are also significant challenges in using CFE for natural product discovery, which we will discuss at specific points through this review (Fig. 1). We will next discuss the historical context of CFE, what constitutes the molecular machinery and reactions of CFE, and different CFE methods currently established. Where possible, we will also highlight similarities between CFE and synthetic chemistry.

### 3.1 Historical context of cell-free expression

CFE has played a major role in the fields of genetics and biochemistry. The pioneering efforts of the Nobel laureate Eduard Buchner (Nobel Prize in Chemistry in 1907) led to the discovery that yeast cell extracts ferment glucose into carbon dioxide and water.^57^ In addition, a major discovery of the 20th century was the unravelling of the genetic code by Marshall Nirenberg, Har Khorana and Robert Holley, which led to a shared Nobel Prize in Physiology or Medicine in 1968.^58–60^ Critical to these experiments were the use of *Escherichia coli* cell-free extracts containing active ribosomes, combined with poly-RNA and single ^14^C-labeled amino acids to initiate protein synthesis. This was remarkable since, at this time, the biochemistry of protein synthesis was unknown. To determine the genetic code, radioactivity was used to assign which DNA triplet (*i.e.*, 64 codons) codes for which of the 20 canonical amino acids. This research was an important stepping stone in the development of modern molecular biology and highlights a key strength of cell-free extracts to study and manipulate gene expression. In another example, Alfred Goldberg and Tom Maniatis determined the mechanism for the ubiquitin-proteasome pathway using yeast CFE.^61,62^ Closer to natural product biosynthesis, Ian A. Scott initially developed the concept of total enzyme biosynthesis using cell-free extracts and purified enzymes to perform complex chemistry, including the biosyntheses of *S*-adenosyl-l-methionine,^63^ vitamin B_12_^64–66^ and taxol.^67^ More recently, this approach has been applied for the biosynthesis of a variety of complex natural products.^68–73^

### 3.2 What is a cell extract?

At their simplest level, cell-free experiments are composed of a cell extract, devoid of a genome, cell wall, and cell membrane. Typically, the extract contains all the proteins, RNA and small molecules required to generate new proteins through transcription–translation, as well as many metabolic enzymes present at the point of cell lysis. Focusing on CFE, this is similar to synthetic chemistry in a broad sense. CFE reactions start from known substrates, and through ribozyme and enzyme-catalysed reactions – some dependent on energy, metals or specialised cofactors to aid catalysis – generate products, often proteins, and sometimes at high yields and efficiency. In contrast to chemistry reactions that can require elevated temperatures, typical CFE models derived from cells grown at mesophilic conditions function best between ∼20–40 °C, although there is a *Thermus thermophilus* CFE model that works at thermophilic temperatures.^74,75^ Finally, in contrast to most synthetic chemistry reactions, CFE does not need high concentrations of expensive or unsustainable metal catalysts, protection/deprotection steps, or toxic organic solvents.

*E. coli* is the dominant CFE model because it is extensively characterised in terms of physiology and multi-omics (*i.e.*, DNA, RNA, protein and metabolite) level characterisation. *E. coli* cells double approximately every 20 minutes when supplemented with rich organic media and saturating oxygen at 37 °C.^76^ Under these conditions, *E. coli* requires less energy for anabolic processes such as making amino acids^77^ or cofactors. About 55% of an *E. coli* cell's biomass is protein,^78^ with ribosome biogenesis requiring up to one-third of a cell's volume or mass.^79,80^ The total number of ribosomes is proportional to cell growth. Fast dividing cells (*T*_D_ = 20 min) contain approximately 70 000 ribosomes per cell.^80^ Slow dividing cells (*T*_D_ > 1 hour) contain less than 8000 ribosomes per cell.^80^ While ribosomes and translational machinery from stationary cells are active,^81^ the most active *E. coli* cell extracts for CFE are typically derived from rapidly dividing cells.

The cell wall and cell membrane are physical barriers that contain a highly crowded molecular mixture of proteins, RNA, and small molecules inside a set volumetric unit – the cell. After cell lysis, the cell extract is less crowded – by between one to two orders of magnitude^52,82–84^ – compared to the inside of a cell. Dividing cells are also continuously making and recycling RNA/proteins. For an *E. coli* cell (∼1 μm^3^) this results in 3–4 million proteins (average size 35 kDa) per cell.^85^ In the cytoplasm there is an estimated total protein concentration of 200–300 mg mL^−1^.^86,87^ Up to one-third of this content is ribosomes, which catalyse their own biogenesis, as well as the cell's proteome. The rest of the cytoplasmic proteins are dedicated to gene expression, metabolism and other processes essential to a cell including structural proteins involved in controlling cell division and cell trafficking.^88,89^ While some of these interactions are well mapped in model organisms such as *E. coli*, their role in molecular crowding and pathway functions remains relatively uncharacterised yet can influence the function of key cell-free enzymatic processes. Examples include reconstituted ribosomes^90^ and enzyme RNA polymerase, whose activity is enhanced by artificial molecular crowding agents in CFE studies.^91^ Other biocatalytic processes can also be stimulated by molecular crowding chemical mimics, including polymers such as polyethylene glycol.^92–95^ However, the rates of activity are less in CFE than within a cell. For example, the ribosomes in CFE translate proteins approximately an order of magnitude slower than inside the cell.^52,87^ This is an important limitation in considering the potential applications of CFE.

Last, engineering living cells to make chemicals can be a challenge. The concentration of metabolites and chemicals spans several orders of magnitude inside a cell.^96,97^ Within any cell type, the optimal balance of single-molecule concentrations is achieved through homeostasis, a process regulated by genetic changes, metabolic flux, and the import and export of chemicals across the cell membrane. This intricate process relies on individually evolved catalytic efficiencies and molecular interactions inherent to each cell type and their associated microenvironment.^97^ Frequently, challenges arise in synthetic biology and metabolic engineering studies when engineering cells to accommodate heterologous enzymes and chemicals. This can lead to disruptions in homeostasis and impairments in cell growth. Each protein has an evolved function within a specific cell type, including how it interacts with other biomolecules (nucleic acids, lipids, proteins and small molecules) and how its own distinct physiochemical properties (*i.e.*, thermostability, solubility limits) control function. Therefore, when placing a heterologous gene/protein into a cell, its expression can lead to various metabolic and genetic imbalances – often generically referred to as “burden”^98–100^ – as well as solubility issues,^101^ leading to stress responses to restore order – *i.e.*, DNA recombination and mutations. The above factors are not relevant for CFE, providing an advantage to this approach.

### 3.3 Cell-free gene expression using *E. coli*

**Fig. 2:**
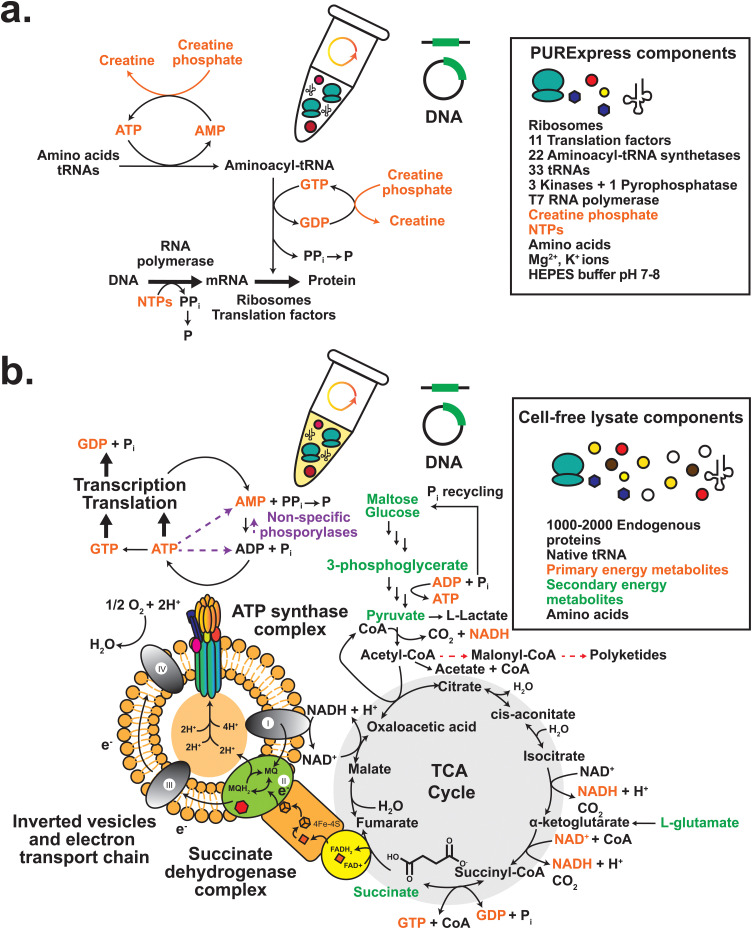
Components and metabolic pathways required for (a) PURExpress® and (b) crude cell extract-based CFE. Metabolic pathways related to cell-free energy regeneration are shown with black solid arrows. Cofactors and primary energy metabolites involved in primary and secondary energy regeneration are coloured orange and green respectively.

A crude cell-free extract has all the essential proteins and RNA required for catalysing gene expression, as well as a variety of anabolic and catabolic metabolic pathways (Fig. 2). There are about 90 proteins, three ribosomal RNA (rRNA) and 33 transfer RNA (tRNA) species required for catalysing CFE.^102,103^ Gene expression begins through expression of DNA by a multienzyme RNA polymerase (RNAP) complex (αβ_1_β_2_ω), which synthesises messenger RNA (mRNA) from the nucleotide bases adenosine triphosphate (ATP), cytosine triphosphate (CTP), guanosine triphosphate (GTP) and thymidine triphosphate (TTP). Specifically, a Sigma factor (*σ*) guides the RNAP holoenzyme to the so-called promoter region of the DNA helix to initiate mRNA transcription, while several other enzymes conduct DNA winding/unwinding. Instead of using the native RNA polymerase, CFE models typically use the heterologous T7 RNA polymerase (derived from T7 bacteriophage – a virus that infects and replicates within *E. coli*) as a high powered and orthogonal alternative. Translation in *E. coli* requires ribosomes, 33 translation factors, 22 aminoacyl-tRNA ligases, 33 tRNA species, 20 amino acids and energy as ATP equivalents. The ribosome is an RNA–protein complex composed of a small 30S (Svedberg units) and large 50S subunit. The 30S subunit requires ribosomal proteins (labelled S1–S12) and both 5S and 16S ribosomal RNA (rRNA). In contrast, the 50S subunit is composed of ribosomal proteins (L1–L21) and 23 rRNA. When total RNA is extracted from actively growing bacterial cells, rRNA and tRNA are the most abundant and stable RNA species, comprising ∼95% of total RNA.^104^ Translation factors are critical for ribosome activity including three initiation factors (IF1–3), three elongation factors (EF-Tu, EF-Ts, and EF-G), three release factors (RF1–3), a ribosome recycling factor (RRF), and the 20 canonical amino acids for coupling to a cognate tRNA. One essential precursory step occurs *via* an aminoacylation reaction catalysed by an aminoacyl-tRNA ligase. Beyond the transcription–translation machinery, there are endo- and exo-nucleases (RNAses) that recycle bulk RNA, as well as peptidases and proteases (∼74 in *E. coli*), chaperones, transcription factors and metabolic enzymes.

Compared to peptide chemical synthesis,^105^*E. coli* ribosomes catalyse amino acid elongation at rates of ∼10–20 amino acids per second.^52^ Atom efficiency and success rate is close to 100%, while incomplete translation products and mRNA transcripts are degraded and recycled back into amino acids and nucleotides.^106,107^ In comparison, solid phase peptide synthesis (SPPS) can be performed at rates of near 10–15 residues per hour^108^ (at most 2.5 residues per minute). SPPS can also make up to 50–100 amino acid polymers with yields approaching 99% for each addition. In terms of limitations, CFE is sensitive to many physical and chemical variables (*e.g.*, pH, temperature, ionic strength). However, there are clear advantages for biological peptide/protein synthesis over SPPS, especially as many proteins are much larger than 100 amino acids in length. In addition, peptides and proteins can be modified post-translationally, and non-canonical amino acids can be incorporated, which is comparable to SPPS. By leveraging transcription/translation however, vast libraries of variable peptide and protein sequences can be generated from DNA templates. Here, CFE provides a unique advantage for making and modifying ribosomal peptides, as discussed specifically in Section 4.1.

In terms of concentration and diversity of proteins in cell extracts, early proteomics studies found about 500–800 different proteins in *E. coli* CFE,^109,110^ while a more recent study (with increased sensitivity) found up to 1892^111^ different proteins – or 43% of what is encoded by the *E. coli* genome. In terms of protein concentration, gene expression proteins are dominant, with elongation factor Tu (EF-Tu) being the most abundant, followed by the ribosomal proteins.^112^ In complement, quantitative mass spectrometry methods have been used to estimate that the ribosome concentration (at 10 mg mL^−1^ total protein) is ∼2.2 μM,^113^ with *E. coli* estimates between 1.6 to 2.3 μM.^52,114^ Individual levels of intracellular proteins were also shown to be sensitive to several physical conditions during cell extract preparation,^111^ including heating and pH/salts. Such information is valuable towards understanding the limitations of CFE and to inform future efforts to improve variability and performance.

Currently, *E. coli* is the most popular cell type for making cell extracts, with the B strains specifically developed as a base for strong heterologous recombinant protein production. During the early 2000s, an *E. coli* derived p̲rotein synthesis u̲sing r̲ecombinant e̲lements (PURE) system was developed from purified components.^115^ This includes purified ribosomes (54 ribosomal proteins) and translation factors, which when combined with T7 RNAP, tRNA, energy regeneration enzymes, substrates (*i.e.*, amino acids, creatine phosphate) and synthetic DNA, reconstitutes transcription–translation within a test-tube.^103,116^ This remarkable engineering feat is commercially available as the PURExpress® kit (New England Biolabs), while another group recently made available a step-by-step protocol and bacterial strains (His_6_-tagged translation factors) for manual preparation of the PURE cell-free reaction.^117,118^ An advantage of PURE is its high efficiency because of an absence of competing side-reactions such as non-specific phosphatases, nucleases and peptidases/proteases, which degrade the energy source, DNA/RNA and proteins. However, limitations of PURE include its high cost and limited potential for scaling up. Several groups have used PURE systems for a variety of applications,^119^ including natural product biosynthesis.^120^

### 3.4 Alternative CFE methods

To provide alternative CFE methods, there is a rising interest in exploiting alternative microbes that offer unique benefits, such as resilience to harsh manufacturing conditions or specific functional advantages – *e.g.*, photosynthesis, minimal cells, natural product hosts. As such, alternative hosts are expected to play an increasingly significant role in synthetic biology.^121^ However, there are several limitations (*e.g.*, genetic tools, characterisation) that hinder wider use. Here, CFE provides an enabling technology to explore such non-model hosts to study gene expression and metabolic reactions. Recent CFE tools from microbes include *Bacillus subtilis*,^122^*Priestia megaterium*,^113^*Pseudomonas putida*,^123^*Vibrio natriegens*,^124–126^*Clostridium autoethanogenum*,^127^ and multiple *Streptomyces* spp.^128–133^ In addition, a related study showed the general flexibility of cell-free methods across several bacterial species and even multi-extract CFE.^134^

The *Bacillus* Gram-positive low G + C genus are traditionally popular in industry because of their strong potential for enzyme and protein secretion, and natural product discovery.^135^ There are two well-established systems for this potential application. First, a *B. subtilis* CFE,^122^ which is derived from a major industrial microbiology model organism. Second, a *Priesta megaterium* CFE (formerly *Bacillus megaterium*) tool, which demonstrated stronger levels of protein synthesis than *B. subtilis* CFE and has minimal protease activity.^136^*P. megaterium* is notable for its industrial use including amylase and vitamin B_12_ production.^136,137^ For future progress, tools that accelerate the development of novel molecular tools are needed for non-model microbes. To meet this need, the *P. megaterium* CFE was used as a rapid prototyping platform for developing gene expression tools, an approach assisted by automation.^136^

Another promising candidate is *Vibrio natriegens*, which has the fastest doubling time of any known cultured microbe at *T*_D_ < 10 min.^138^*V. natriegens* is also compatible with *E. coli* plasmids and genetic systems.^138–140^ Three groups reported the development of *V. natriegens* CFE tools, providing high protein yields (up to 1.6 mg mL^−1^) equivalent to *E. coli* CFE.^124–126^ The *V. natriegens* CFE was also active with linear DNA templates with protection from degradation by the Cro DNA-binding protein.^141^

Finally, the high G + C Actinomycetota phylum, which includes the *Streptomyces* genus, remain the most studied in terms of natural products isolated and characterised. Over 3000 strains have been catalogued, and many are slow-growing and/or challenging to genetically engineer. For this, *Streptomyces* CFE from different models has been developed for studying gene expression or pilot expression studies.^128–133^ Examples of their initial uses for natural product discovery will be highlighted within the review, while a more focused review on *Streptomyces* CFE is available.^37^

### 3.5 Cell-free metabolism

Early CFE studies were based on *in vitro* translation (IVT) reactions, when an RNA template was added to a cell extract with ATP as the primary energy source. However, the native energy pool and extract associated ATP consuming phosphorylases are a key limiting factor for extended mRNA and protein synthesis in these early IVT/CFE methods. Crude cell extracts are dynamic in terms of their ability to catalyse anabolic and catabolic metabolic reactions.^113,142,143^ There are hundreds of metabolic enzymes present in the cell extract dependent on the growth condition and processing steps used for extraction,^111^ suggesting that cell-free extracts have the unrealised potential to be engineered to catalyse more complex chemical reactions. CFE can make energy as ATP equivalents from a secondary energy source. Through catabolism of a high-energy carbon substrate, CFE will generate reducing equivalents *via* primary metabolism, which have the potential to couple to other biosynthetic pathways *via* redox active proteins (*e.g.*, Fe–S) and protein-bound cofactors, such as nicotinamide adenine dinucleotide (NAD), flavin adenine dinucleotide (FAD) and mononucleotide (FMN). In synthetic chemistry, rare metal catalysts such as palladium are often used to perform equivalent reduction reactions. Another advantage that CFE provides over living cells is the facile integration of synthetic enzyme pathways through the addition of purified enzymes or mixing of different cell-free extracts and enzymes, also referred to a cell-free metabolic engineering.^144–147^ This enables rapid prototyping, as well as the use of non-natural precursors (*i.e.*, non-canonical amino acids) and sacrificial substrates such as polyphosphate or phosphite for energy regeneration such as ATP^148^ and NADH/NADPH^149,150^ recycling *via* kinase and dehydrogenase enzymes, respectively.

Focusing on energy sources, several CFE studies initially used creatine phosphate/kinase-based systems for energy regeneration.^41,151,152^ Recent research has since identified a range of high energy carbon substrates as an alternative energy source to regenerate ATP within the cell extracts.^113,143,153–155^ These energy sources are categorised as primary or secondary. The primary energy sources (∼0.5–3 mM) are the nucleotides ATP, GTP, CTP and TTP (or diphosphate and monophosphate equivalents), which are utilised as the building blocks for mRNA synthesis in transcription. ATP/GTP then has an additional role as energy for translation: 1 molar equivalent of ATP is required to initiate translation, 2 for activation of the amino acids to form a single aminoacyl-tRNA, 2–3 for peptide elongation, and 1 for translation termination by a release factor protein (RF1–3).^156,157^ The secondary energy source (∼10–50 mM) is required to regenerate the primary energy source (∼1–2 mM) that initially powers mRNA synthesis, while some ATP/GTP is used to begin protein synthesis and in charging the aminoacylated-tRNA species. Therefore, most CFE reactions use a working concentration of ATP/GTP higher than CTP and TTP.^158^ The secondary energy source then generates ATP equivalents through catabolism of a high-energy carbon substrate. This occurs because the extracts contain the metabolic enzymes and complete pathways for these reactions. While glucose can be used as an energy source in CFE,^142,143,159^ other high-energy substrates from the glycolysis pathway such as pyruvate,^160^ phosphoenolpyruvate,^161^ 3-phosphoglycerate,^162^ and l-glutamate^163^ provide more direct sources of energy (Fig. 2). Interestingly, *E. coli* cell-free extracts also contain inverted vesicles. Early studies established that CFE synthesis of chloramphenicol acetyltransferase is oxygen-dependent and disrupted by known electron transport chain (ETC) inhibitors.^164^ These vesicles are an artefact of cell lysis and contain parts of the ETC and ATP synthase, also observed by proteomics.^111,165^

A limitation of cell-free extracts is the presence of many non-specific phosphorylase enzymes, which degrade the bulk nucleotide triphosphate (NTP) pool and therefore contribute to overall energy loss in CFE. Calculations highlight up to 98% of total energy is wasted in cell extract CFE.^113^ Here, advancements in machine learning have assisted with modelling and prediction of “winning” combinations of this multi-parameter space in CFE.^166^ In addition, there are background metabolic enzymes and pathways that can be activated to contribute to energy production. For example, l-glutamate, an amino acid and nitrogen donor, can provide extra ATP. l-Glutamate enters the Krebs cycle *via* a transaminase reaction to generate α-ketoglutarate. Uniquely, *P. megaterium* CFE was active with succinate as a secondary energy source,^113^ presumably catabolised *via* the succinate dehydrogenase complex to feed into to the ETC.

Cell-free extracts also contain a range of amino acid, nucleotide, fatty acid, and sugar metabolites, whereas in PURE all starting substrates and cofactors are controlled for. In crude cell-free extracts, the addition of exogenous NAD^+^ and coenzyme A can improve CFE productivity,^52,158^ suggesting these cofactors are rate-limiting in cell-free extracts. Together with energy and reducing equivalents, NAD and coenzyme A are important cofactors or coenzymes for secondary metabolism. For example, acetyl-CoA, malonyl-CoA and related analogues are substrates for polyketide biosynthesis.

## 4. CFE for biosynthesis

In previous sections, we explored the distinct features of CFE and its potential uses for biosynthesis. Now, we will delve into specific types of natural products and other chemical products engineered using CFE. We will refer to this broader approach as cell-free biosynthesis (CFB), or cell-free transcription–translation and biosynthesis. For instances where there are gaps in the literature, we will highlight similar approaches using cell-free extracts or purified enzymes. We will also highlight key advantages and limitations of CFE/CFB for specific chemical classes or related biomolecules where applicable.

### 4.1 CFB of RiPPs

**Fig. 3:**
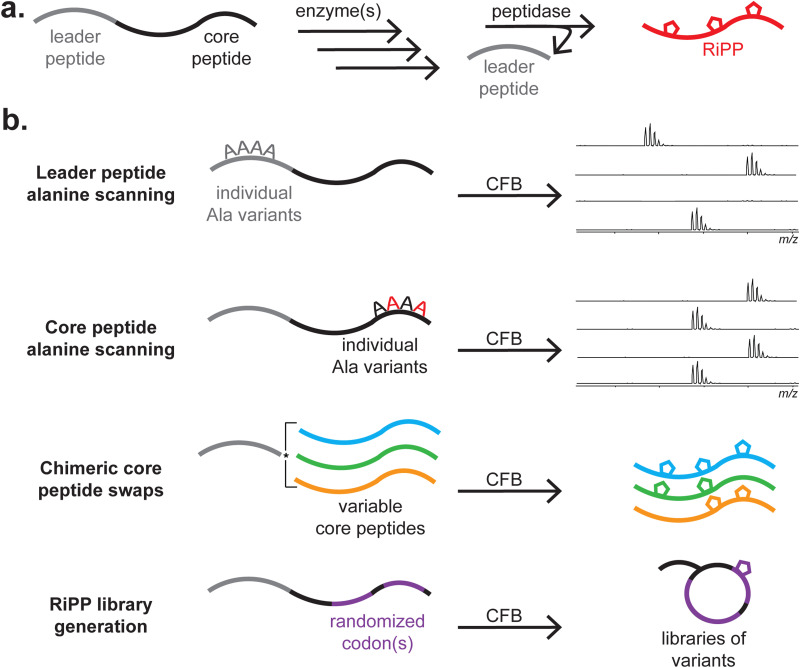
Summary of RiPP biosynthesis. (a) A generalised schematic of RiPP biosynthesis, in which one or several enzymes act on a precursor peptide to form a mature RiPP. (b) Several experiments in which CFB can be leveraged are depicted.

The ribosomally synthesised and post-translationally modified peptides (RiPPs) family are a well-suited class of natural products for CFB (Fig. 3 and Table 1). This is because the structure and function of RiPPs are directly amendable at the level of the DNA sequence. RiPPs are translated as an inactive precursor peptide, often consisting of an N-terminal leader region involved in substrate binding and a C-terminal core region modified by one or several enzymes.^167^ Proteolysis of the leader peptide often results in the mature RiPP, although further tailoring modifications can occur. By separating the sites primarily responsible for recognition and modification, Nature exploits this as a strategy for library generation. As RiPPs are DNA encoded, variants of the final product can be readily produced by mutagenesis of the precursor peptide gene. However, generating extensive libraries of precursor peptides is laborious, and there is no guarantee that any given modifying enzyme will accommodate these variations in substrate. Furthermore, variants produced by heterologous expression are limited to the 20 standard amino acids unless amber stop codon suppression, chemical modification, or another strategy is used.^168,169^ SPPS is an option, but this is a relatively low-throughput strategy, and most precursor peptides are long enough that cost, peptide solubility, and secondary structure can be problematic.

**Table 1:** Examples of RiPP expression in CFPS systems

Year(s)	RiPP class	Biosynthetic enzyme(s)	RiPP(s) generated	Cell-free strategy	Metabolite yield(s)	Ref.
2022	Pyritide	MroB, MroC, MroD	Pyritide A1 and analogs	PURExpress	Not generated	(Nguyen *et al.*, 2022)^170^
2020	Pyritide, thiopeptide	LazB, LazC, LazD, LazE, LazF	Lactazole and analogs	FIT-Laz	Not generated	(Vinogradov *et al.*, 2020)^171^
2021, 2023, 2025	Lasso peptide	FusB, FusC, FusE	Fusilassin and analogs	*E. coli* BL21 (DE3) lysate	Not generated	(Si *et al.*, 2021),^172^ (Kretsch *et al.* 2023),^173^ (Barrett *et al.* 2025)^174^
2021	Lasso peptide	BurB, BurC, BurD	Burhizin	*E. coli* BL21 (DE3) lysate	Not generated	(Si *et al.*, 2021)^172^
2021	Lasso peptide	CapB, CapC, CapD	Capistruin	*E. coli* BL21 (DE3) lysate	∼40 μg mL^−1^	(Si *et al.*, 2021)^172^
2021	Lasso peptide	CelB, CelC, CelE	Celulassin	*E. coli* BL21 (DE3) lysate	Not generated	(Si *et al.*, 2021)^172^
2021	Lasso peptide	FusB, FusC, FusE	Halolassin	*E. coli* BL21 (DE3) lysate	Not generated	(Si *et al.*, 2021)^172^
2007	Lanthipeptide	NisB, NisC	Nisin	*In vitro* rapid translation system, supplemented with ZnSO_4_	∼200 IU per mL	(Cheng *et al.*, 2007)^175^
2020	Lanthipeptide	NisB, NisC, NisP	Nisin and analogs	*E. coli* BL21 (DE3) lysate, supplemented with ZnCl_2_	∼180 IU per mL	(Liu *et al.*, 2020)^176^
2024	Lanthipeptide	SboM, SboT	Salivaricin B and analogs	UniBioCat, derived from *E. coli* BL21 Star (DE3) Δ*degP* Δ*pepN* lysate	Not generated	(Liu *et al.*, 2024)^177^
2022	Circular bacteriocin	N/A	Garvicin ML	PURExpress, SICLOPPS	Not generated	(Peña *et al.*, 2022)^178^
2020	Pantocins	PaaA	Pantocin A and analogs	FIT system	Not generated	(Fleming *et al.*, 2020)^179^
2021	Pyrroloquinoline quinones	N/A	PQQ	*Gluconobacter oxydans 621H* cell lysate	Not generated	(Wang *et al.*, 2021)^180^
2019	Proteusin	N/A	*N*-Methylated Nhis-AerA	*Microvirgula aerodenitrificans* cell lysate	Not generated	(Bhushan *et al.*, 2019)^181^

CFB enables the rapid assessment of each residue's potential contributions to substrate recognition and modification status (Fig. 3). Additionally, core peptides can often be swapped out to generate novel natural products, and the production of vast libraries of precursor peptides has been readily achieved. Variants can be generated in a high-throughput manner by several methods, including error-prone polymerase chain reaction (PCR) and site saturation mutagenesis.^182,183^ This library can then be assayed against the modifying enzyme(s), often without additional purification steps. In some examples, both the precursor peptide and modifying enzymes have been expressed through CFB, resulting in the *de novo* cell-free production of RiPP natural products directly from DNA.^172^ Orthologous enzymes could be assayed this way without expressing and purifying each individually. However, modifying enzymes are primarily purified separately *via* affinity chromatography and added to the reaction to conserve resources for CFB of the precursor peptide. For several uses of CFB in studying the bioactivity of RiPPs, we direct the reader to a recent review on RiPP mechanisms of action.^184^

#### 4.1.1 Application of the flexizyme system to ribosomal peptides

In recent years, the flexizyme system was developed to incorporate non-proteinogenic amino acids in *E. coli*, expanding the peptide chemical space. Briefly, the flexizyme system consists of *de novo* ribozymes that can charge a wide variety of non-native amino acids. The flexizyme recognises the 3′-end of tRNA (RCCA-3′ where R = G or A as the discriminator base at position 73) as well as a benzylic or cyanomethyl ester moiety on the leaving group, and catalyses tRNA aminoacylation.^185–188^ By establishing a distinct recognition site, the system can tolerate a wide variety of tRNAs and aminobenzyl esters as the aminoacyl-acceptor and amino-acyl donors. These includes α-l-amino acids, α-*N*-methyl l-amino acids, α-*N*-acyl l-amino acids, α-d-amino acids, β-amino acids, and α-hydroxy acids, permitting a broad range of peptide scaffolds to be generated. When coupled with an *in vitro* system, the flexizyme system is referred to as “flexible *in vitro* translation” (FIT).

As an example of the flexizyme system's usage, a platform for assessing the percentage of peptide natural products accessible by CFB was devised.^189^ By parsing data from several online repositories, the authors developed a chemoinformatic resource for data on both linear and macrocyclic peptide natural products. Leveraging this data, they chose 43 peptides to express in either PURExpress® or PUREfrex, most of which (36, ∼84%) were successfully produced. Cyclisation of 14 of 31 head-to-tail macrocyclised candidates was achieved using either the macrocyclase PatG_mac_ from the patellamide biosynthetic pathway or PCY1 from the orbitide biosynthetic pathway. Flexizymes were successfully utilised to incorporate a highly represented piperazic acid motif and *N*-methyl, d- and β-amino acids, although deficiencies are often observed during their incorporation.^190^ Overall, this work provides a wealth of peptide natural product data and highlights potential areas of prioritisation in the CFB production of peptide natural products, including RiPPs.

**Fig. 4:**
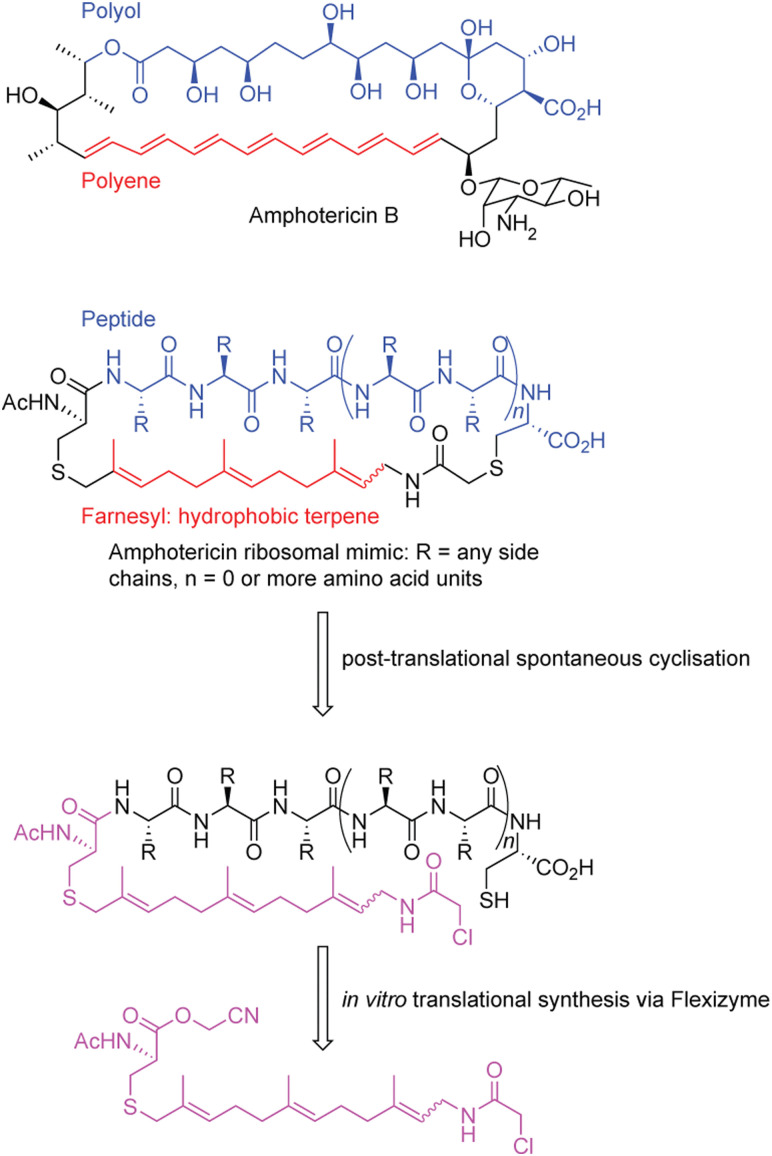
Generation of amphotericin-like peptide analogues with the flexizyme system.^191^

In a separate example, a macrocycle was generated that was inspired by amphotericin B, an antifungal polyketide metabolite that has activity that depends on both a hydrophobic polyol region as well as a lipophilic polyene region^191^ (Fig. 4). Importantly, amphotericin, despite being an incredibly valuable antifungal, displays high levels of renal-toxicity because of sterol sequestering in humans and in fungal pathogens.^192^ Thus, developing compounds that retain bioactivity, but decrease its toxicity are desired. Despite amphotericin lacking an overall peptide scaffold, the researchers devised a peptide with general mimicry of amphotericin B's physiochemical features.^191^

To construct a molecule resembling amphotericin, the authors used a peptide backbone to mimic the polar polyol portion and a terpene farnesyl region to mimic the polyene. To generate their structure, an S_N_2 reaction is leveraged between an N-terminal chloroacetamido moiety at the end of the farnesyl group, incorporated *via* FIT, and a cysteine residue in the peptidic portion.^193^ While structurally impressive, the activity of this compound is unexplored. Overall, the above examples show the utility of FIT to create natural products and natural product inspired molecules *via* synthetic biology.

#### 4.1.2 Pyritides and azole-containing RiPPs

Pyritides are a macrocyclic class of RiPPs.^194^ Pyritide biosynthesis consists of the glutamyl-tRNA-dependent dehydration, *via* MroB and MroC, of Ser and possibly Thr to form dehydroalanine (Dha) and sometimes dehydrobutyrine (Dhb).^72,195^ The enzymatic macrocyclisation of the precursor peptide defines the class.^196^ A pyridine synthase, MroD, links two Dha moieties through a formal [4+2]-cycloaddition to form a six-membered nitrogenous heterocycle. In most cases, subsequent dehydration and aromatisation with concomitant removal of the leader peptide form the mature RiPP.^197^

CFB has been used to assess the substrate scope of the MroBCD enzymes involved in the biosyntheses of pyritide A1 and A2 from *Micromonospora rosaria*. An array of substrates was rapidly evaluated using commercially available PURExpress®. MroBCD exhibited a wide substrate tolerance, including macrocycle expansion and contraction, suggesting that pyritide enzymes may be suitable for future engineering.^170^ Notably, recognition of both an N-terminal leader sequence and the C-terminal tripeptide “WLI” on MroA2 by MroB and MroD explain the broad substrate promiscuity shown between these regions of the precursor peptide.

Almost all pyritides are also thiopeptides, which feature additional modification and frequently possess potent antibacterial activity.^198,199^ Besides a pyridine or dehydropiperdine, thiopeptides contain azol(in)e heterocycles formed from Ser, Thr, and/or Cys residues. Core biosynthetic steps were first elucidated using the enzymes from the thiomuracin BGC, found in *Thermobispora bispora*.^72,195^ An ATP-dependent YcaO cyclodehydratase (TbtFG) phosphorylates the peptide backbone and uses the amino acid residue side chain for nucleophilic attack to generate a 5-membered heterocycle, which is often dehydrogenated by a FMN-dependent enzyme (TbtE).^200^ Azole formation typically precedes the installation of additional PTMs in biosynthesis.^72,195^

**Fig. 5:**
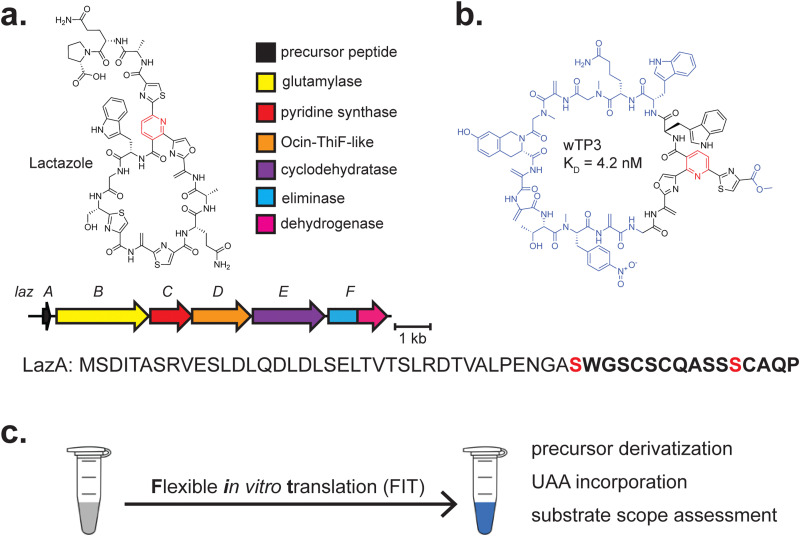
Thiopeptides. (a) BGC and structures of lactazole. The class-defining PTM is highlighted in red. The core peptide is bolded, and the residue(s) on which class-defining PTMs occur are red. (b) The structure of wTP3, a pseudo-natural product developed through years of extensive work using LazB-F. The regions of the compound that differ from lactazole are highlighted in blue. (c) Stylised workflow visualisation for the Flexible *in vitro* Translation (FIT) system, in which the components for transcription/translation, DNA, flexizyme(s), and unnatural amino acid(s) are combined to achieve the goals stated to the right.

CFB has been extensively leveraged in work on the thiopeptide lactazole to develop pseudo-natural products that could serve as drug candidates^201,202^ (Fig. 5a and b). While thiomuracin biosynthesis had been studied previously,^72,195^ it remained to be seen if the proposed order of biosynthetic events was conserved in other thiopeptide BGCs. CFB was successfully leveraged to explore the substrate scope of most enzymes in the lactazole BGC^171^ (LazA-F) (Fig. 5a). LazA was produced through a flexible *in vitro* translation (FIT) system (FIT-Laz),^203^ consisting of purified ribosomes and necessary components for transcription-coupled translation *in vitro*^204^ (Fig. 5c). The FIT system has previously been used for CFB of azoline-containing peptides and dissection of goadsporin biosynthesis, all of which are linear.^205,206^ Various combinations of purified biosynthetic enzymes were added to assess activity on LazA and elucidate any cooperativity between the enzymes.^171^ While thiomuracin biosynthesis can be cleanly delineated into several steps, LazA-F act in a more complex and nuanced manner. Leveraging the capacity for unnatural amino acid installation, the authors incorporated cycloleucine and several backbone *N*-methylated amino acids into a mature thiopeptide and assessed which regions of LazA are amenable to insertion, deletion, and substitution. The FIT-Laz system circumvented the requirement for intensive cloning, heterologous expression, and purification of the assessed substrates.

This work proved foundational for future engineering efforts with lactazole fueled by CFB. A site saturation mutagenesis library was generated for LazA and assessed using mRNA display, in which a translated peptide is joined to its reverse-transcribed coding sequence *via* puromycin to covalently link phenotype with genotype.^207,208^ The authors used a precursor peptide library with N-terminal biotinylation generated using FIT, as well as a C-terminal human influenza hemagglutinin (HA) affinity tag and puromycin linker. After modification by LazB-F (Fig. 5a), HA affinity purification removes cleaved leader peptide from the reaction mixture. An additional streptavidin purification separates modified products from unmodified products in which the leader peptide and biotin groups are attached.^209^

After using mRNA display to assess the full scope of LazB-F modification, the authors evaluated the precise substrate requirements for each modification.^210^ Using numerous LazA variants, they determined the order of biosynthetic events and found that, rather than formation of azoles first and Dhas second as in thiomuracin biosynthesis, lactazole biosynthesis intertwines these steps. Again, the use of CFB to generate these precursor peptides streamlined this process. The FIT-Laz system was then leveraged to explore the substrate promiscuity of glutamate elimination domains such as LazC, finding the enzymes to be highly substrate promiscuous, as was suspected previously.^211^ CFB and mRNA display have also been coupled with machine learning to assess substrate preferences for LazBF and LazDEF.^212^ In this approach, next-generation sequencing was used to generate vast quantities of processing data on substrates. A relatively simple machine learning approach was employed in which peptides were represented as extended-connectivity fingerprints.^213,214^ These data were used to train a deep convolutional neural network^215^ in assessing the potential suitability of substrates. Despite utilizing ∼10^7^ samples for training this model, the preferences for both groups of enzymes remain complex, highlighting the need for high-throughput testing of variable precursor peptides.

Most recently, LazB-F have been used to generate *de novo* thiopeptide pseudo-natural products with nanomolar affinity to Traf2- and NCK-interacting kinase (TNIK), an established target in several cancers.^201,216^ This was achieved by using a similar approach to previous mRNA display work^209^ but including an additional step in which counter-selection against immobilised TNIK enriches for binders to the targeted kinase. Leveraging of a minimised genetic code resulted in the selection of another TNIK binder with a similar affinity to their previous compound (nM) but improved serum stability and incorporation of several unnatural amino acids^201^ (Fig. 5b). This strategy could, in principle, be applied to generate thiopeptides with affinity to any desired immobilised target. This has recently been achieved to evolve binders to both interleukin-1 receptor-associated kinase 4 (IRAK4), an important component of Toll-like receptor signaling,^217^ and the TLR10 cell surface receptor, which exhibits unique anti-inflammatory activity.^218,219^ Overall, the ability to assess extensive libraries of variants (>10^12^) in a proven directed-evolution workflow has enabled an unprecedented understanding of this biosynthetic pathway's potential and streamlined the selection of designer pseudo-natural products.

Alternative strategies for thiopeptide intermediate generation have also been employed. In the production of thiocillin and lactazole, flexizymes have been used to incorporate phenylselenocysteine, which undergoes oxidative elimination with H_2_O_2_ to generate Dha.^220^ This circumvented the need to use a glutamyl-tRNA-dependent dehydratase (such as TbtB or LazB) in producing Dha, which is required for thiopeptide production. While other chemical methods to generate Dhas exist, such as dehydrothiolation of Cys,^221^ this method often conflicts with the activity of azole-forming YcaO enzymes (such as TbtG and LazE), which typically act first in biosynthesis and may cyclise the target Cys residues.^195^

Streptolysin S (SLS) belongs to the linear azole-containing peptide class of RiPPs. SLS is a critical virulence factor in the human pathogen *Streptococcus pyogenes*.^222^ In the biosynthesis of SLS, *S. pyogenes* and other organisms use a putative protease SagE. To assess if SagE truly is a leader peptidase, *in vitro* transcribed and translated precursor peptide SagA, featuring ^35^S labeling, was supplied to *S. pyogenes* lysate.^223^ The proteolysis of SagA supported the putative role of SagE, and additional experiments established this protease is critical for SLS production and showed inhibition by FDA-approved HIV protease inhibitors.

#### 4.1.3 Lasso peptides

**Fig. 6:**
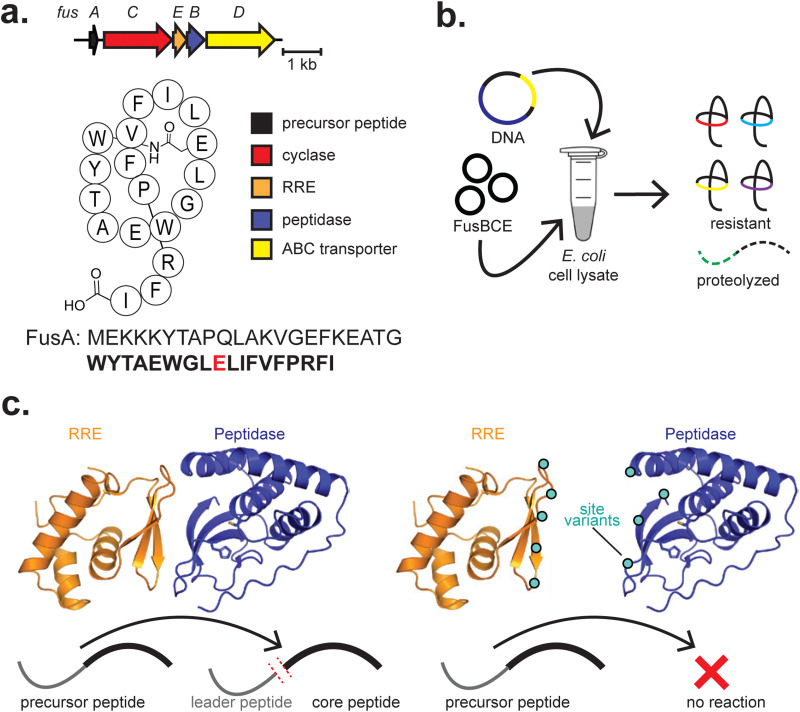
Lasso peptides. (a) BGCs and structures of fusilassin. The class-defining PTM is highlighted in red. The core peptide is bolded, and the residue(s) on which class-defining PTMs occur are red. (b) Stylised workflow visualisation for lasso peptide derivatisation by CFB. The necessary components (DNA and biosynthetic enzymes) are added to clarified *E. coli* lysate to produce lasso peptides and native proteases digest unmodified precursors. (c) An allosteric interaction between an RRE (FusE) and a leader peptidase (FusB) leads to successful leader peptide cleavage in fusilassin biosynthesis. Site variants of the RRE–peptidase interface, depicted as cyan circles, can disrupt the protein–protein interaction, preventing leader peptide cleavage.

CFB has also been used to study lasso peptides. Lasso peptides are kinetically trapped [1]rotaxanes which feature a macrolactam installed *via* a lasso cyclase, providing them with high thermal stability and protease resistance^224^ (Fig. 6a). Lasso peptide biosynthesis consists of leader peptide proteolysis, followed by isopeptide bond formation in a threaded conformation supported by the lasso cyclase and further maintained by bulky residue(s) in the “tail” region.^225^ Recent work extensively leveraged cell-free methods to bolster *in vitro* characterisation methods.^172^ Primarily using *E. coli* cell extracts prepared in-house, several lasso peptides were produced by introducing DNA encoding the precursor peptides and respective biosynthetic enzymes. While cell lysate includes endogenous proteases that will degrade the unmodified precursor peptide, cyclised lasso peptides are highly resistant (Fig. 6b). The substrate scope of the lasso cyclase FusC was then assessed, finding that this enzyme is relatively tolerant to single-site replacements throughout the FusA precursor peptide core sequence and multi-site replacements in the “ring” region (residues 2–6). This study is also an example of a novel RiPP, halolassin, being discovered using CFB.^172^ While the biosynthetic enzymes from a similar BGC were used in its production (FusBCE of fusilassin biosynthesis), the product structure is as expected given the native enzymes. We expect CFB-mediated discovery of RiPPs to be a relatively straightforward approach and attribute the lack of current examples to underutilisation.

In subsequent work on lasso peptides, site-directed mutagenesis and cell-free production of the fusilassin protease (FusB) and precursor recognition (FusE) enzymes were critical in revealing an allosteric interaction between the two^173,180^ (Fig. 6c). While FusE variants could be readily produced by PURExpress, robust production of FusB variants was only achieved in *E. coli* cell extracts, highlighting the complementary use of different CFE platforms.^173^ In the extract, crowding effects and chaperones may have potentially contributed to successfully producing FusB variants, though any precise mechanism has not yet been elucidated. Cell-free production of numerous protein variants expedited testing, and additional experiments corroborated the results. Both studies highlight the potential for cell-free production of enzyme site variants in assessing complex biosynthetic pathways and protein–protein interactions.

Recently, interactions between the FusA precursor peptide and FusC have also been assessed.^174^ Cell-free generation of both FusA and FusC variants was utilized, in combination with molecular dynamics simulations and bioinformatic analysis, to explore how FusC interacts with FusA. This work further highlights the utility of CFB in generating variants of both a RiPP enzyme and its peptide substrate.

#### 4.1.4 Lanthipeptides

CFB has also been used in the study of lanthipeptides. Lanthipeptides are a class of RiPPs in which Cys and Dha/Dhb are covalently tethered through a Micheal-like addition reaction to form (methyl)lanthionine cross-linked peptides, often consisting of several macrocycles.^226^ They frequently display potent antibacterial activity, among other activities,^184^ and are a relatively well-studied class of RiPPs. In an early case of CFB in the study of RiPPs, the lantibiotic (lanthipeptide antibiotic) and food preservative, nisin, was successfully reconstituted in a commercial *E. coli in vitro* Rapid Translation System.^175^ Both the precursor peptide and modifying enzymes were supplied as DNA, and additional zinc sulfate was provided for the zinc-dependent lanthipeptide cyclase NisC.^227^ The authors confirmed successful nisin production *via* western blot with a nisin A-specific antibody and activation of a nisin-inducible promoter, *P*_nisF_, to generate green fluorescent protein (GFP).^175^

In a separate example, a Zn^2+^ supplemented *E. coli* cell extract was used to produce nisin and several genome-mined lanthipeptides.^176^ All 18 uncharacterised nisin analogs in the National Center for Biotechnology Information (NCBI) database were identified. Genes encoding the core peptides of these RiPPs were then fused C-terminally to the nisin Z leader peptide gene sequence, and the resulting chimeric precursors were subjected to NisBC modification. Six peptides showed dehydration, and four were shown to have antibacterial activity. This work further emphasises the breadth at which predicted RiPPs can be generated with CFB. Saturation mutagenesis was used to generate a nisin variant, M5, with improved antibacterial activity against *E. coli*. CFB mixture was co-incubated with a growing *E. coli* culture to assess antibacterial activity. This approach circumvents potential cytotoxicity issues from the production of fully modified nisin *in vivo* and exemplifies the utility of CFB in producing cytotoxic RiPPs.

Recently, a unified biocatalysis (UniBioCat) system for the rapid engineering of lanthipeptides was achieved.^177^ This strategy differs from previous work in that all components are generated *via* CFB, including the precursor peptide, modification enzyme(s), requisite protease(s), and transporter(s). Using this UniBioCat system, they first biosynthesized the lanthipeptide, salivaricin B from *Streptococcus salivaris* K12 in an *E. coli*-derived lysate. Further, they utilized a modified *E. coli* lysate with several proteases knocked out to screen both salivaricin B and 46 analogues. After establishing proof of concept, the UniBioCat system was used to express uncharacterized lanthipeptides identified *via* genome mining. Similarly to previous work on lasso peptides,^172^ the core peptide genes of uncharacterized lanthipeptides were fused to the salivaricin precursor peptide (SboA) leader for modification by its cognate lanthipeptide cyclase SboM. This represents further advancement of the ability to use RiPPs for CFE and highlights the potential to generate all requisite components for biosynthesis in a single CFB reaction (Table 1).

#### 4.1.5 Other CFB applications for RiPPs

Numerous other RiPP classes have also benefited from cell-free methods. For example, several circular bacteriocins, including garvicin ML, have been produced in PURExpress.^178^ Circular bacteriocins are a relatively unadorned class of RiPPs that feature N- to C-terminal macrocyclisation.^228^ Using the split-intein mediated method for ligation of peptides and proteins (SICLOPPS),^229^ the authors produced a peptide that, when non-enzymatically cyclised, forms mature garvicin ML. This approach obviated the need for the native macrocyclisation enzyme and potentially broadened the sequence space that can be used. Such an approach could be broadly used for head-to-tail macrocyclised peptides.

**Fig. 7:**
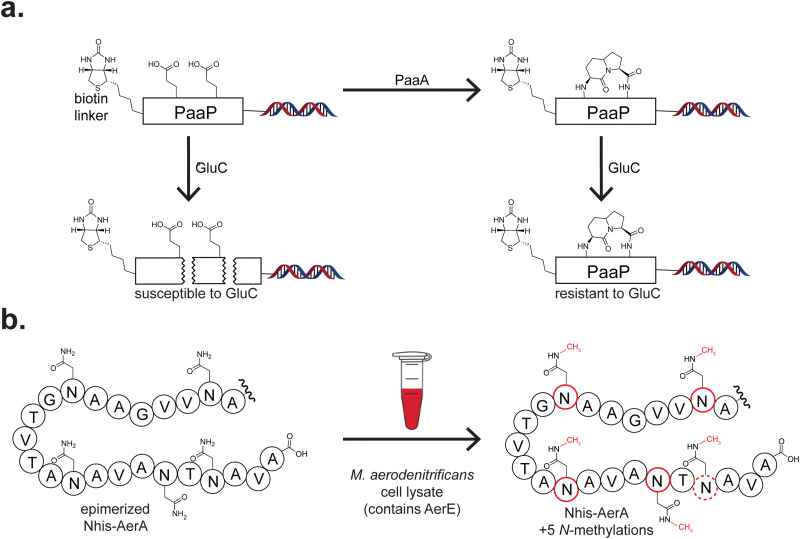
Additional examples of cell-free biosynthesis in RiPPs. (a) The structure of the modified precursor peptide PaaP is derived from two glutamate residues. Once modified by the enzyme PaaA, PaaP is resistant to GluC cleavage, providing a selection mechanism for modified variants of PaaP. (b) Supplying *M. aerodenitrificans* lysate with pure Nhis-AerA co-expressed with AerD in *E. coli* led to five side-chain *N*-methylations, represented by the red outlines and CH_3_ groups. The dashed outline on Asn43 indicates that methylation was not directly observed but proposed based on additional MS fragmentation data.

Display technologies have also been frequently used with CFB. The biosynthetic scope of PaaA, an enzyme catalysing two dehydration/decarboxylation reactions on two Glu residues of the precursor peptide PaaP to form pantocin A (Fig. 7a), has been explored through the FIT system coupled with mRNA display.^179^ The authors use endoproteinase GluC cleavage to assess substrate modification, rapidly sorting viable substrates from non-substrates (Fig. 7a). To validate hits from their screening, they produced several precursor variants using PURExpress and then assessed *in vitro* activity. In a single set of experiments, they glean insight into the core recognition and enzyme promiscuity of PaaA.

Several other examples of CFB use lysates from the native producers, including production of the RiPP pyrroloquinoline quinone (PQQ). PQQ is a redox cofactor used by several bacterial dehydrogenases.^230^ PQQ biosynthesis occurs mostly in Pseudomonodota, although other microbes incapable of its production can scavenge exogenous PQQ.^231,232^ Precursor peptide PqqA was heterologously expressed and purified, then modified in cell lysate from a strain that naturally produces PQQ, *Gluconobacter oxydans* 621H.^233^ This strategy may prove useful for scaling up the production of RiPPs with robust biosynthetic enzymes.

A unique strategy was employed while screening for the production of a predicted proteusin product, aeronamide.^181^ Proteusins are a heavily modified class of RiPPs defined by an unusually long leader peptide composed of a bundle of alpha-helices, and were initially bioinformatically predicted before their isolation and structural elucidation.^167,234,235^ These compounds feature extensive epimerisation and methylation, exhibiting antibacterial activity through the formation of ion channels.^236–239^ To confirm the expression and activity of the remaining enzymes in the aeronamide BGC, researchers spiked epimerised, partially modified precursor peptide AerA into lysate (Fig. 7b). They observed a mass shift indicative of five expected substrate methylations.^181^ Because lysate from the native producer *Microvirgula aerodenitrificans* was used for this experiment, the authors confirmed active enzymes in their culturing conditions before scaling up production. As RiPP precursor peptides and their expected mass shifts due to modification are often predictable, we envision this being a beneficial strategy.

While most uses feature the addition of DNA to generate proteins of interest, several examples feature the use of *in vitro* transcription to generate biosynthetically crucial tRNA. Reconstitution of NisB and TbtB, dehydratase enzymes involved in lanthipeptide and thiopeptide biosynthesis, respectively, has required glutamyl-tRNA^Glu^.^72,240^ In these cases, the tRNA was generated first as a DNA template and then transcribed using RNA polymerase. tRNA^Glu^ was glutamylated either separately or concurrently with NisB/TbtB activity *in vitro*. Further *in vitro* characterisation of tRNA-dependent RiPP enzymes will likely benefit from a similar strategy for tRNA generation.

A similar enzyme is responsible for pearlin biosynthesis. In pearlins, a peptide aminoacyl-tRNA-ligase (PEARL) enzyme elongates the C-terminus of a precursor peptide by a single amino acid residue in a tRNA-dependent manner.^241^ PEARLs are structurally related to tRNA^Glu^-dependent dehydratases. In studying the biosynthesis of 3-thiaglutamate by the PEARL TglB, modified TglA-derived substrates were generated by PURExpress.^242^ This enabled further characterisation of downstream modifying enzymes TglHI.

#### 4.1.6 Outlook for ribosomal peptides

Looking forward, we predict the broader use of CFB in the discovery, study, and engineering of RiPPs and other ribosomal peptides. In RiPP discovery, screening for native production of a novel predicted RiPP in cell extract would decrease culture scale and cost, allowing for a more comprehensive array of screening parameters. Purified precursor peptide could also be spiked into the extract, amplifying the signal and providing orthogonal evidence that a predicted mass is, in fact, the desired RiPP intermediate or product. One could even envision coupling such strategies with high-throughput refactoring of BGCs for the discovery of bioactive RiPPs.^243,244^

While broadly applicable for a substrate scope assessment, CFB is often performed qualitatively. Frequently, a significant excess of enzyme relative to substrate is provided in each reaction, and reaction times may be long. When assayed non-quantitatively, potentially poor substrates can be more effectively modified. Recent work on the lactazole BGC supports this, with a more narrow window of preferred substrates than modified substrates.^218^ It should, therefore, be stressed that while some precursor peptides may be tolerated in CFB, they may be poor substrates when assayed under more stringent conditions. In short, while any given enzyme may modify a substrate, this method does not inform whether this modification is necessarily efficient.

Most RiPP CFB studies use PURExpress. Reasons for this include ease of use and much higher replicability when compared to preparing *E. coli* cell lysate for CFE in-house. Endogenous proteases in cellular extract pose a significant issue in RiPP biosynthesis. Several classes of RiPPs, including pyritides, lasso peptides, and lanthipeptides, are highly resistant to proteases because of their macrocyclic structures. This resistance can provide a selection mechanism for modified precursor peptides.^172^ For protease-susceptible classes of RiPPs, additional measures must be employed to use cell lysates from *E. coli* and other bacteria fully. Unidentified components in these lysates may assist in the folding and stability of biosynthetic enzymes,^44^ but a direct mechanism has yet to be identified.

CFB will also expedite RiPP engineering, which may involve assaying millions of substrate variants for processing and activity. Designer substrates can readily interrogate complex pathways' biosynthetic order of events.^171^ Individual components can be included, excluded, or altered to assess interactions between RiPP-modifying enzymes. Numerous classes of RiPPs have not yet been extensively characterised, and could be, in an approach like that taken with the lactazole pathway. As has been discussed, both RiPPs and other ribosomal peptides can also be tailored using the FIT system to develop pseudo-natural products that improve upon Nature's designs. As cell-free methods are optimised and lysate sources beyond *E. coli* are more widely used, we predict the study of RiPPs will benefit greatly.

### 4.2 CFB of non-ribosomal peptides

**Fig. 8:**
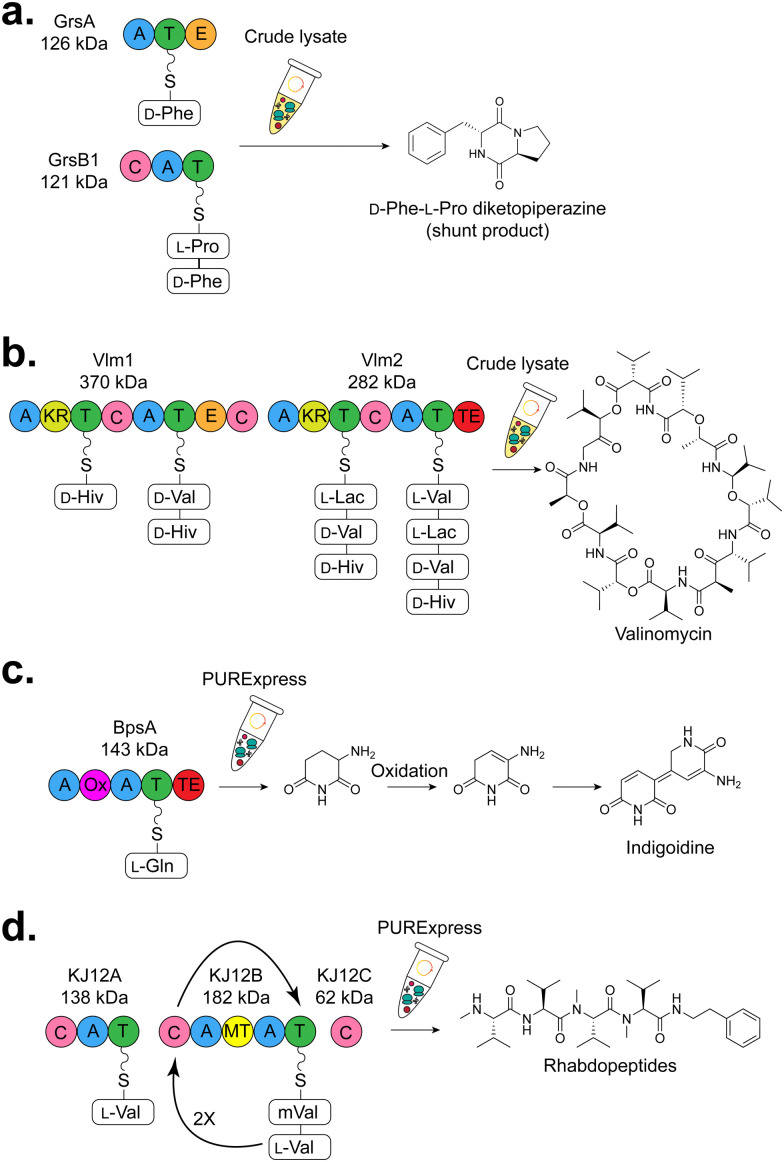
Overview of CFB of NRPS products (a) GrsA and GrsB1, the first couple modules from the gramicidin S NRPS were used to generate a d-Phe-l-Pro diketopiperize product.^263^ (b) Valinomycin NRPS, the largest NRPS enzyme and metabolite produced by CFE to date.^267^ (c) Blue pigment synthetase A (BpsA) which generates the blue pigment indigiodine.^264^ (d) Rhabdopeptide-like peptide (RXP) from *Xenorhabdus* KJ12.1.^264^ Abbreviations: A = adenylation, T = thiolation, C= condensation, Ox = oxidase, TE = thioesterase, MT = methyltransferase, KR = ketoreductase, E = epimerase.

Although both generate peptides, the biosynthetic logic behind RiPPs and nonribosomal peptides (NRPS) is distinct, though there is an overlap in their overall scaffolds *via* convergent evolution to form bioactive moieties. For example, both non-ribosomal peptides and RiPPs frequently have numerous *N*-methyl and d-amino acids, and heavily oxidised and/or cross-linked side chains.^167^ Recently, acylated RiPPs, including lipoavitides^245^ and selidamides,^246^ have been discovered that have similarities to non-ribosomal lipopeptides such as the polymyxins,^247^ and daptomycin.^248^ This is suggestive that regardless of biosynthetic machinery, using predicted and known structures from peptide natural products is a fruitful area for bioactive discovery. Applications of CFB to investigate non-ribosomal peptides (Table 2) have been pursued both using the PURE system and crude lysates.^249^ NRPS are composed of a series of modular domains that select building blocks (with NRPS, amino acids) that are elongated on a phosphopantetheinylated carrier protein in a colinear fashion.^250–252^ Briefly, NRPS consist of an adenylation (A) domain that recognises the amino acid and activates the amino acid monomer at the C-terminus as an adenylate followed by transfer to a peptidyl carrier protein (PCP) sometimes known as a thiolation (T) domain. The activated, loaded amino acid is condensed *via* the C-domain. Optional domains such as epimerase (E), cyclisation (Cy), methyltransferase (MT) and oxidase (Ox) domains generate structural variety. Chain release typically occurs *via* hydrolysis or macrocyclisation by a thioesterase (TE) domain (Fig. 8),^253–255^ though in some systems a reductase (R) domain reduces the product to a primary alcohol^256,257^ (Fig. 8). Additional tailoring may occur on the carrier protein and the released metabolite. One of the attractive features of NRPS is that non-proteinogenic amino acids can be incorporated without the use of flexizymes, genetic reprogramming, or further tailoring (*e.g.* as occurs with modifying enzymes in RiPPs). This modularity is attractive from a synthetic biology perspective, and determining the best junctions for chimeric and selectivity swapped pathways has been an area of considerable attention recently.^258–262^

**Table 2:** Examples of NRPS expression in CFPS systems

Year(s)	NRPS gene(s)	Non-ribosomal peptide metabolite(s)	Cell-free strategy	Metabolite yield(s)	Protein yield(s)	Ref.
2017	GrsA and GrsB1 from *Brevibacillus brevis*	d-Phe-l-Pro diketopiperazine (DKP), shunt product of gramicidin S	*E. coli* BL21 Star (DE3) lysate	∼12 mg L^−1^	GrsA ∼106 μg mL^−1^	(Goering *et al.*, 2017)^263^
					GrsB1 ∼77 μg mL^−1^	
2020	GrsA from *Brevibacillus brevis*	Gramicidin S	PURExpress	Not generated	Not quantiated	(Siebels *et al.*, 2020b)^264^
2020	Vlm1 and Vlm2 from *Streptomyces tsusimaensis*	Valinomycin	*E. coli* BL21 Star (DE3) lysate and cell-free protein synthesis-metabolic engineering (CFPS-ME)	∼30 mg L^−1^	Not quantitated	(Zhuang *et al.*, 2020)^265^
2020	BpsA from *Streptomyces lavenduale*	Indigiodine	PURExpress	∼62 μg mL^−1^	BpsA: ∼28.7 μg mL^−1^	(Siebels *et al.*, 2020b)^264^
2023	BpsA from *Streptomyces lavenduale*	Indigiodine	*E. coli* BL21 Star (DE3) lysate	∼223 μg mL^−1^	BpsA: ∼95 μg mL^−1^	(Dinglasan *et al.*, 2023)^266^
2020	KJ12A, KJ12B, KJ12C from *Xenorhabdus* KJ12·A	RXP (rhabdopeptide-like peptides)	PURExpress	Not quantified	Not quantified	(Siebels *et al.*, 2020b)^264^
2020	IndC from *Photorhabdus luminescens*	Indigiodine	PURExpress	Not generated	Not quantified	(Siebels *et al.*, 2020b)^264^
2020	TycB from *Bacillus brevis*	Tyrocidine	PURExpress	Not generated	Not quantified	(Siebels *et al.*, 2020b)^264^
2023	TycA from *Bacillus brevis*	Tyrocidine	*E. coli* BL21 Star (DE3) lysate	Not generated	Not quantified	(Dinglasan *et al.*, 2023)^266^
2023	Pys from *Pseudomonas entomophilia*	Pyreudione	*E. coli* BL21 Star (DE3) lysate	Not generated	Not quantified	(Dinglasan *et al.*, 2023)^266^
2021	TxtA and TxtB from *Streptomyces scabiei*	Thaxtomin A	*Streptomyces venezuelae* ATCC 10712 lysate	Not generated	Not quantified	(Moore *et al.*, 2021)^132^
2021	NH08_RS0107360 from *Streptomyces rimosus*	Uncharacterized	*Streptomyces venezuelae* ATCC 10712 lysate	Not generated	Not quantified	(Moore *et al.*, 2021)^132^

#### 4.2.1 NRPS from gramicidin S

The first successful CFB of a non-ribosomal peptide was a cyclic dipeptide d-Phe-l-Pro diketopiperazine (DKP).^263^ DKP is a naturally occurring shunt product found in the early modules of the gramicidin S biosynthetic pathway^268^ and has commonly been used as a miniaturised model system for NRPS engineering (*e.g.*, domain swapping^259^ and A-domain characterisation^269^). The antibiotic gramicidin S is produced by *Brevibacillus brevis* through two NRPS enzymes: GrsA (126 kDa, one module) and GrsB (510 kDa, four modules). The isolated combination of GrsA and the first module of GrsB (GrsB1) forms DKP *via* a spontaneous intramolecular cyclisation process.^268^ As a first proof of concept, an *E. coli* lysate-based platform for CFB of d-Phe-l-Pro DKP was used.^263^ To ensure adequate phosphopantetheinylation of the carrier proteins, the commonly used promiscuous PPTase, Sfp from *Bacillus subtilis*, was used along with a fluorescently labelled Bodipy-CoA substrate to screen for adequate post-translational modification. 12 mg L^−1^ DKP was produced by CFB, which is comparative to titres with cell-based biosynthesis (9 mg L^−1^). This work established proof of concept that active NRPS pathways can be reconstituted with CFB (Fig. 8A).

#### 4.2.2 Valinomycin

Rather than just a small model shunt product, other examples have leveraged entire NRPS BGCs in CFB. The 36-membered cyclododecadepsipeptide valinomycin was selected, which is naturally synthesised by various *Streptomyces* strains^270–272^ and possesses a wide range of bioactivities.^273^ The valinomycin biosynthetic pathway requires two NRPS proteins, Vlm1 (370 kDa, two modules) and Vlm2 (282 kDa, two modules). The priming unit for the A-domain of the first module is α-ketoisovalerate (Kiv) which is subsequently reduced to d-2-hydroxyisovalerate (d-Hiv) by a ketoreductase domain (KR). The A and the epimerase (E) domain of the second module activated and converted the l-Val to d-Val. Likewise, in the third module, activated pyruvate (Pyr) is reduced by a KR domain to l-lactate (l-Lac); and last, l-Val is activated in module 4. Valinomycin was synthesised, oligomerised and macrolactonised *via* a TE domain of three tetradepsipeptide scaffolds of d-α-Hiv-d-Val-l-Lac-l-Val (Fig. 8b).^267^ The first approach used *E. coli* BL21 Star (DE3) lysate to synthesise Vlm1 and Vlm2 for valinomycin synthesis, and the *B. subtillis* Sfp in a single-pot reaction. This co-expression was successful; however, the titre of valinomycin was low (∼9.8 μg L^−1^). To optimise this reaction, addition of a type II thioesterase (TE-II)^274^ increased titres 4-fold to ∼37 μg L^−1^. Here, lysates for cell-free extracts were harvested from cells making the target proteins. CFB synthesis of valinomycin was initiated from two lysates enriched with Vlm1 and Vlm2 proteins, respectively. Because of the low titre (∼5.5 μg L^−1^), further optimisation was investigated including supplementing cofactors (CoA, NAD, and ATP), and optimising the ratio of Vlm1 and Vlm2 lysates. Ultimately, valinomycin titre was increased to 77 μg L^−1^ without cofactor supplementation and with a mass ratio of 3 : 1 (cell lysate-Vlm1 : cell lysate-Vlm2). To increase valinomycin production, TE-II was added using a two-step approach. TEII was first produced by CFE and then added to the CFB reaction of Vlm1 and Vlm2. This single change increased valinomycin production 375-fold to 29 mg L^−1^. As glucose is the key reaction substrate, further optimisation of glucose concentrations gave the highest valinomycin titre at ∼30 mg L^−1^ (200 mM glucose). Taken together, this is the first example of a whole natural product NRPS gene cluster of valinomycin (>19 kb) made using CFB. The yield of valinomycin improved with the addition of an associated editing enzyme (TE-II).

#### 4.2.3 Indigoidine and rhabdopeptides in the PURExpress system

Beyond cell extract based CFB, the PURExpress® system has also been used for some model NRPS pathways. This includes blue pigment synthetase A (BpsA) from *Streptomyces lavendulae*, and RXP (rhabdopeptide-like peptide) from the bacterium *Xenorhabdus* KJ12.1.^264^ BpsA is a single module NRPS that catalyses the biosynthesis of indigoidine from two l-glutamine substrates *via* an A domain, oxidation (Ox) domain, and TE domain (Fig. 8c). To generate the BpsA holoenzyme using the PURExpress® system, Sfp from *B. subtilis* was added to the reaction. BpsA titre was ∼15.5 μg mL^−1^ when co-expressed with Sfp, and ∼28.7 μg mL^−1^ in a two-step reaction. Other NRPSs that were screened for synthesis by the PURExpress® system include IndC (a BpsA homolog from *Photorhabdus lumincens*), GrsA, TycB1 from tyrocidine biosynthesis, and the *Xhenorhabdus* KJ12.1 KJ12ABC. The RXP-synthesizing NRPS KJ12ABC from the bacterium *Xhenorhabdus* KJ12.1 was investigated in the PURExpress® system as an example of a more complex target. The BGC of RXP encodes three NRPS modules, *kj12A* (encoding a C-A-T module, 137.8 kDa), *kj12B* (encoding a C-A/MT-T module, 181.5 kDa), and *kj12C* encoding a stand-alone C-_terminal_ domain (62.3 kDa). Three peptides were identified with the molar ratio of 10 : 1.5 : 1 (KJ12A : B : C): mV-V-mV-mV-PEA, V-V-V-mV-PEA, and V-mV-V-mV-PEA (*N*-methylated valine (mV), valine (V), and phenylethylamine (PEA)) (Fig. 8d). The yields of rhabdopetides were not quantitated, and only relative ratios of proteins were reported. This work shows the utility of applying the PURExpress® system directly to generate natural products from genomic DNA.

#### 4.2.4 CFB of single module NRPSs with *E. coli* CFE

The optimisation of the lysate and energy mix composition of CFE is typically performed using fluorescent proteins;^275^ however, biosynthetic proteins such as NRPSs are substantially different from GFP in terms of their structural characteristics and composition. The synthesis of megasynthase proteins with CFE is a significant challenge because of RNA degradation, premature ribosome termination, and resource demands on the supply of energy and amino acids. All of this collectively results in issues with full length NRPS synthesis when using^263,265,276^ CFB.^277^ In prior studies, low throughput methods such as denaturing polyacrylamide gels and/or MS-based proteomic evaluation^263,265^ were used to assess the success of NRPS synthesis using CFE. To increase throughput, a tetracysteine (TC) peptide tag fused to the C-terminus of the NRPS protein was used to detect protein synthesis, through binding of the TC disulphide to a fluorogenic organo-arsenic dye.^266,278^ Various reaction conditions were optimised for the CFB production of indigoidine including the precursor l-glutamine, coenzyme A, and plasmid DNA, which resulted in ∼900 μM indigoidine (∼3.6-fold higher than the yield reported in the PURExpress® system).^264^ This detection method furthers opportunities to investigate and optimise parameters that could constrain the synthesis and activity of NRPS proteins, such as cofactor requirements (*e.g.*, magnesium ion), precursor availability (*e.g.*, amino acids), and catalytically active A domains that consume stoichiometric ATP. Various concentrations of PEP, ATP, magnesium ions, and amino acids were also tested for BpsA-TC, BpsA-TC with an inactive A domain (BpsA E315A), and superfolder GFP (sfGFP) to examine the impact of catalytic activity of the A domain.^266^ These experiments indicate that (1) functional CFE of NRPS modules requires additional input due to the catabolism of ATP by the active NRPS A domain, (2) the active A domain impacts NRPS CFE Mg^2+^ requirements, and (3) standard CFE conditions used for fluorescence protein synthesis are not ideal for NRPS expression.

Ultimately, the reporter-based system determined reaction conditions that were more optimal for BpsA than initial conditions optimised on GFP. These optimised reaction conditions can be used for other monomodular NRPSs with similar architectures to BpsA: TycA (124 kDa), and Pys (142 kDa). The result demonstrated that with these two enzymes, the improvement in expression compared to ‘standard CFE conditions’ was even more dramatic than the improvement in BpsA expression (∼1 order of magnitude). Further, this work reveals the importance of optimizing beyond simple fluorescent protein reporters when using CFE approaches.

#### 4.2.5 Synthesis of NRPS proteins using *Streptomyces* CFE

Beyond *E. coli*, *Streptomyces* CFE has been used to produce NRPS enzymes. *Streptomyces* is known as a major source of numerous clinically relevant NRPS-derived natural products including bleomycin,^279^ vancomycin,^280–282^ and daptomycin,^283,284^ which is a driver for developing highly active *Streptomyces*-based CFE for rapid prototyping.^37^ Initial efforts to develop a *Streptomyces* CFE platform as a tool for biological investigation were made in the 1980s.^285^ Following that, a high-yielding *Streptomyces* CFE protocol using a *Streptomyces lividans* system was established.^131^ Several commonly used *Streptomyces* strains were tested for this CFE model using eGFP at first, then high G + C (%) content genes from Actinomycetota. Besides optimisation of GFP, tailoring proteins from the nonribosomal peptide tambromycin were generated (*tbrP*, *tbrQ*, *tbrN*) along with the type II thioesterase from the valinomycin biosynthetic pathway. While accessory genes to non-ribosomal peptide biosynthesis as opposed to NRPSs themselves, this still sets a precedence for *Streptomyces*-based CFE platforms to produce high G + C genes from biosynthetic pathways.^131^ A separate effort to develop a *Streptomyces*-based CFE platform used fluorescent proteins to optimise the genetics (*e.g.*, promoter) and reaction conditions,^129,133^ such as the ATP source, and RNase inhibitor, with the goal of developing a specific energy solution. Next, high G + C content genes were used to test this system, including NRPS enzymes such as the thaxtomin A biosynthesis proteins from *Streptomyces scabiei* (TxtA, TxtB,^132^ TxtD and TxtE^286^), and an uncharacterised NRPS from *S. rimosus*.^132^ Challenges that *E. coli* CFE faces, such as codon bias, solubility issues, complexity, and lack of post-translational modifications^132,287^ can be circumvented using this system. Considering the high GC-content genes of most NRPS enzymes, *Streptomyces*-based CFE have proven to be more suitable as CFE hosts compared to *E. coli*.^131^

### 4.3 CFB of polyketides

To our knowledge, there is a single example of a detectable amount of a polyketide metabolite being produced by CFE.^288^ However, there have been several examples of polyketide synthases (PKSs) being generated in cell-free studies, which along with *in vitro* studies utilising purified proteins, are suggestive of future promise for applying CFE to generate polyketide metabolites (Table 3).

**Table 3:** Examples of PKS expression in CFPS systems

Year(s)	PKS gene(s)	PKS type	Associated polyketide metabolite	Cell-free strategy	Metabolite generated?	Protein yield(s)	Ref.
2017	DEBS1-TE (first gene from the 6-deoxyerythronlide B fused to the thioesterase)	Type I	Erythromycin	*E. coli* BL32 Star (DE3) lysate *E. coli* TB3 lysate	Not generated	Not quantified	(Hurst *et al.*, 2017)^276^
2020	PikAIII (5th module from the pikromycin PKS)	Type I	Pikromycin	PURExpress	Not generated	Not quantified	(Siebels *et al.*, 2020b)^264^
2020	DEBS module 4	Type I	Erythromycin	PURExpress	Not generated	Not quantified	(Siebels *et al.*, 2020b)^264^
2024	RppA	Type III	Flaviolin	*E. coli* BL32 Star (DE3) lysate	Flaviolin generated, not quantified	Not quantified	(Sword *et al.*, 2024)^288^

**Fig. 9:**
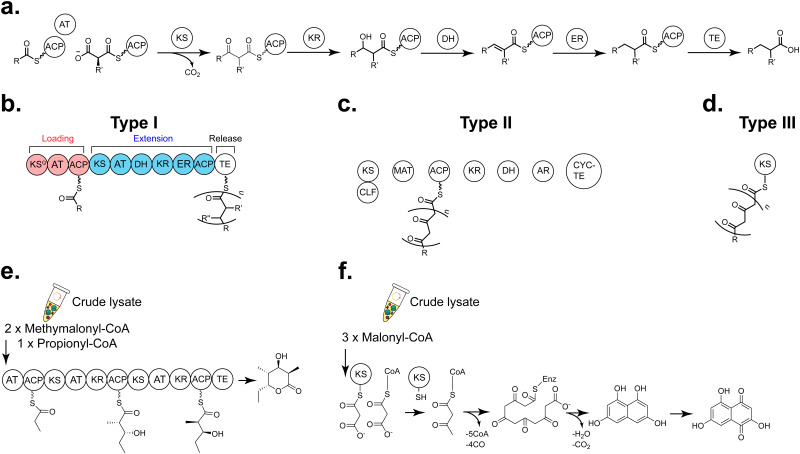
CFB of PKS products. (a) Enzymatic reactions catalysed by PKS domains. (b) Type I PKSs consist of all domains fused as one “megasynthase” and either can be modular in the case of bacteria (one round of extension and β-carbon processing per module) or iterative (multiple rounds of β-carbon processing per module) in the case of fungi. β-Carbon processing domains (KR, DH, and ER) are optional. (c) Type II PKSs consist of the domains as discrete enzymes. A KS-chain length factor (CLF) is responsible for condensation, a malonyl acyl transferase (MAT) extends and a cyclase TE (CYC-TE) is responsible for cyclisation of the aromatic products. β-Carbon processing domains (KR and DH) are optional. (d) Type III PKS acts upon a CoA substrate rather than an acyl carrier protein in an interative fashion. (e) DEBS-1 TE, the first gene in the 6-deoxyerythronolide B synthase (DEBS) fused to the thioesterase, consisting of a loading module and two extension modules, 275 KDa which was generated in an *E. coli* cell-free lysate. (f) RppA, the type III PKS which generates the red brown polyketide synthase, flaviolin which was generated in an *E. coli* cell-free lysate. Abbreviations: ACP = acyl carrier protein, AT = acyltransferase, KS = ketosynthase, KR = ketoreductase, DH = dehydratase, ER = enoylreductase, TE = thioesterase.

Polyketides are a family of diverse and complex natural products that possess chemical architectures with broad-ranging bioactivity. Polyketide biosynthesis consists of sequential condensations of malonyl-CoA or malonyl-CoA derivatives in a fashion analogous to fatty acid biosynthesis, albeit with a greater variance of reductive processing of the β-keto moiety. Canonical PKSs consist of the following enzymatic domains: a ketosynthase (KS) that is responsible for condensation of malonyl-CoA and derivatives thereof in a Claisen-like fashion, and an acyltransferase (AT) which selects an extender unit and condenses it to an acyl carrier protein (ACP) (Fig. 9). Scaffolds are additionally modified by optional beta carbon processing domains, including a ketoreductase (KR), which reduces the keto group stereoselectively to a hydroxy group, a dehydrase (DH), which dehydrates to form an olefin, and an enoylreductase (ER), which forms a saturated carbon backbone. Often, the polyketide is terminated by a thioesterase which typically either hydrolyses from a linear product or macrolactonises.^289,290^

Polyketide synthases are divided into three sub-classes: type I, type II, and type III. Type I PKSs have a multi-domain architecture that is similar to the mammalian fatty acid synthase, wherein domains with discrete enzymatic activity are covalently linked and are the prototypical megasynthases.^291–295^ Type I PKSs from bacteria are typically modular, where there is one elongation per module (set of condensations followed by subsequent reduction) and are further categorised as *cis*-AT and *trans*-AT PKSs depending on whether the acyltransferase domain is embedded or present on a separate domain.^296–298^ The type I modular PKSs are found in Actinomycetota and Myxobacteria^298^ whereas *trans*-AT PKS are found in a broad range of bacterial phyla.^299^ Type I fungal PKSs^296,300–303^ and some other eukaryotic PKSs^304^ have a similar multi-domain architecture, but catalyse the elongation of the polyketide chain in an iterative fashion. Type II PKSs are almost exclusively found in soil and marine bacteria, traditionally Actinomycetota, but have recently been shown to occur in other phyla. Type II PKSs have domains organised into discrete proteins that dock to a KS/chain length factor (KS/CLF) complex, and traditionally form non-highly reductive products, such as oxytetracyclines.^305–308^ Type III PKSs are found in plants,^309,310^ fungi,^300^ and bacteria^311^ and function on standalone CoA substrates and typically form chalcone-like scaffolds.^312,313^ Many iterations of polyketide pathways exist outside of these paradigms, including fusions with other natural product classes^314,315^ (PKS/NRPS^316^ or PKS/terpene hybrids^317^), unusual domains^318–324^ and irregular substrate selectivity,^325^ rendering PKSs one of the most fascinating targets for construction of diverse carbon scaffolds *via* bioengineering.

#### 4.3.1 Type I PKSs

The application of CFE for modular polyketide synthase design is especially promising. Type I PKSs and collinearity between domain composition and order and metabolite structure has presented clear potential genes to molecules *via* any route that can be envisaged through PKS biosynthetic logic. As well as their applications to generate therapeutics, synthetic biology efforts have also applied type I PKSs to generate bioproducts including adipic acid,^326^ hydroxy acids,^327–329^ short-chain ketones,^330^ and delta lactones.^331^ Evolutionary approaches^332–334^ such as chemoinformatics,^329^ and homology-based rational domain exchange^325–327^ have all shown some success in domain replacement. However, the design-build-test-learn cycle remains challenging because of the large size of these enzymes.^335–337^ Successful *in vitro* biosynthesis of type I polyketides include the erythromycin PKS, more commonly known as 6-deoxyerthronolide synthase (DEBS), using crude lysates from *Streptomyces coelicolor*.^338^ This generated “triketide lactone” small molecules resulting from three condensations of methylmalonyl-CoA in *E. coli* lysate.^339,340^ In addition, the production and isolation of a silent polyketide from *Norcardia puris* isolates^341^ was obtained using purified proteins expressed in *E. coli*. Despite this potential, there are still significant challenges to making functional megasynthases with CFE. Therefore, the initial focus has been to make apo-proteins. For this there has been limited progress. Doktycz and coworkers used proteomics to explore complex protein synthesis in cell-free lysates^276^ by investigating the first module of the 6-deoxyerythronolide B synthase fused to the TE (DEBS1-TE) expressed in *E. coli* BL21 (DE3) CFE co-expressing the promiscuous phosphopantetheinyltransferase Sfp^342^ (Fig. 9e). This “mini-synthase” is commonly used as a model PKS for enzymology studies and consists of a loading module and the first two modules of the erythromycin PKS fused to the TE resulting in a six membered lactone.^343–346^ While post-translationally modified holo-ACPs could be detected, DEBS was largely C-terminally truncated, suggesting that significant transcriptional and/or translational falloff was occurring.^276^ This is consistent with what is observed *in vivo* for type I PKSs in *E. coli*, suggesting that *E. coli* protein synthesis is sub-optimal for the synthesis of megasynthase proteins. Further efforts to use lysate-based CFE that might be more amenable to expression, such as *Streptomyces* sp. CFE, remains an area for exploration.^130–133,347^

PURExpress® has also been explored for generating type I PKSs, however, only protein synthesis has been achieved. A selection of type I PKS proteins including the 14th module from the rapamycin PKS (RAPS module 14), the 5th module from the pikromycin PKS (PikAIII), and DEBS module 4, have been generated.^264^ Besides PKS proteins, four variations of the murine fatty acid synthase (FAS) were generated, which shares architectural similarity to type I PKSs. In all cases, successful labelling with a fluorescent mimic of CoA (CoA-647) was observed, demonstrating they were likely correctly folded as the ACP domain could be modified, suggesting promise for *in vitro* catalytic activity.^264^

#### 4.3.2 Type II PKSs

Historically, heterologous expression of type II polyketide metabolites in tractable hosts has been challenging, potentially due to difficulties with protein synthesis and folding of the KS-CLF homodimer, as well as stoichiometry imbalances.^308,348–352^ Here, CFE provides an opportunity to refactor genetic parts controlling type II PKS expression. While there are not yet any examples of a type II PKS pathway completely reconstituted by CFE, *S. venezuelae* CFE was used to produce several enzymes from the oxytetracycline type II PKS pathway.^64,65^ Separately, there are examples of reconstituting type II PKS pathways with purified enzymes and extracts. These include the oxytetracycline intermediates using both *E. coli* and *Streptomyces coelicolor*^353^ and the enterocin pathway from *Streptomyces martmarimus*.^70,71^ Besides biosynthesis, here there is a link to using CFE to probe antibiotic mechanism of action, with some of the earliest studies of CFE used to study tetracycline back in 1966.^354^ A focused discussion on CFE for bioactivity-guided natural product discovery is provided in Section 5, along with their role for studying antibiotic resistance and mechanisms.

#### 4.3.3 Type III PKSs

To date, the only polyketide completely produced by CFE comes from the type III PKS family.^288^ Type III PKSs are simpler than other types of PKSs, as they typically lack reductive domains and use free malonyl-CoA substrates, so they do not require a phosphopantetheinyltransferase. An example of a bacterial type III PKS was applied to generate flaviolin, a red-brown pigment catalysed by RppA from *Streptomyces griseus*.^288^ Here, *E. coli* BL21 Star (DE3) CFE was used to profile different RppA codon optimised variants, exploring gene expression regulation (Fig. 9f). In addition, the product of the reaction, flaviolin, provided a semi-quantitative reporter (absorbance at 340 nm). This allowed the overall reaction to be optimised by fine-tuning the substrate malonyl-CoA levels (between 5–500 μM). This data suggested that free malonyl-CoA was rate-liming in CFE^288^ and the need for cofactor regeneration to support the engineering of polyketide biosynthesis. While not using CFE, others showed up to 100% conversion of l-tyrosine into raspberry ketone – a high value fragrance and a type III PKS natural product – using a mixture of plant, fungal, and bacterial purified enzymes.^355^ In contrast, microbial cell production of raspberry ketone has poor titres due to the general antimicrobial effects of this compound.^356^ Related fragrance chemicals (non-polyketides) have also been rapidly optimised using CFE, such as limonene.^145^ Here, because of their smaller size and minimal complexity, the type III PKS enzymes are a more immediate target for cell-free synthetic biology, such as pathway refactoring or engineering and replacement of plant type III PKS enzymes with bacterial homologues.^310^

#### 4.3.4 Prospects for CFB and engineering of PKSs

Polyketide synthases are immensely attractive scaffolds for engineering the condensation of malonyl-CoA and malonyl-CoA like units. Key issues to consider in optimising CFB for applications in PKS engineering include limitations of protein size (in the case of type I PKS), determining appropriate cofactor balance (particularly of malonyl-CoA) and post-translational activation by phosphopantetheinylation. Further, the ability to prototype operon design *via* cistronic structure and stoichiometry of discrete gene products in type II would be highly valuable.

### 4.4 Terpenes

Unlike PKS or NRPS biosyntheses, terpene biosynthesis consists of discrete terpene synthases that polymerise and isomerise isoprene units (C_5_H_8_)_*n*_ as dimethylallyl pyrophosphate (DMAPP) and isopentenyl pyrophosphate (IPP), two structural isomers. These isoprene units are produced by two distinct metabolic pathways: the mevalonate (MVA) pathway and the non-mevalonate (MEP) pathway. After these units are polymerised, they are further rearranged, oxidized, and reduced. Terpenes form an immense amount of the biodiversity observed in microbial and plant biosynthesis and are bioactive compounds (*e.g.* the anticancer agent taxol, the antimalarial artemisinin), lubricants, flavourants, and fragrances. Both the biosynthesis of monomers and the chemistry done to the isoprenoid scaffold require substantial quantities of cofactors. The titres of terpenes are typically low to moderate depending on application (<0.5 g L^−1^ up to ∼1.5 g L^−1^) with a few exceptions such as amphoadiene which can be produced at 40 g L^−1^ in *Saccharomyces cerevisiae* (Table 4).

**Table 4:** Examples of terpene generation in CFPS systems

Year(s)	Terpene	Cell-free strategy	Metabolite yield	Ref.
2016	Limonene	*E. coli* BL32 Star (DE3) lysate	60 μg L^−1^	(Dudley *et al.*, 201 6)^146^
2020	Limonene	*E. coli* BL21 Star (DE3) lysate, iPROBE	610 mg L^−1^	(Dudley *et al.*, 2020)^145^
2020	Bisabolene	*E. coli* BL21 Star (DE3) lysate, iPROBE	1010 mg L^−1^	(Dudley *et al.*, 2020)^145^
2020	Pinene (mixture of α- and β-pinene)	*E. coli* BL21 Star (DE3) lysate, iPROBE	Not reported	(Dudley *et al.*, 2020)^145^

#### 4.4.1 Limonene, pinene, and bisabolene

**Fig. 10:**
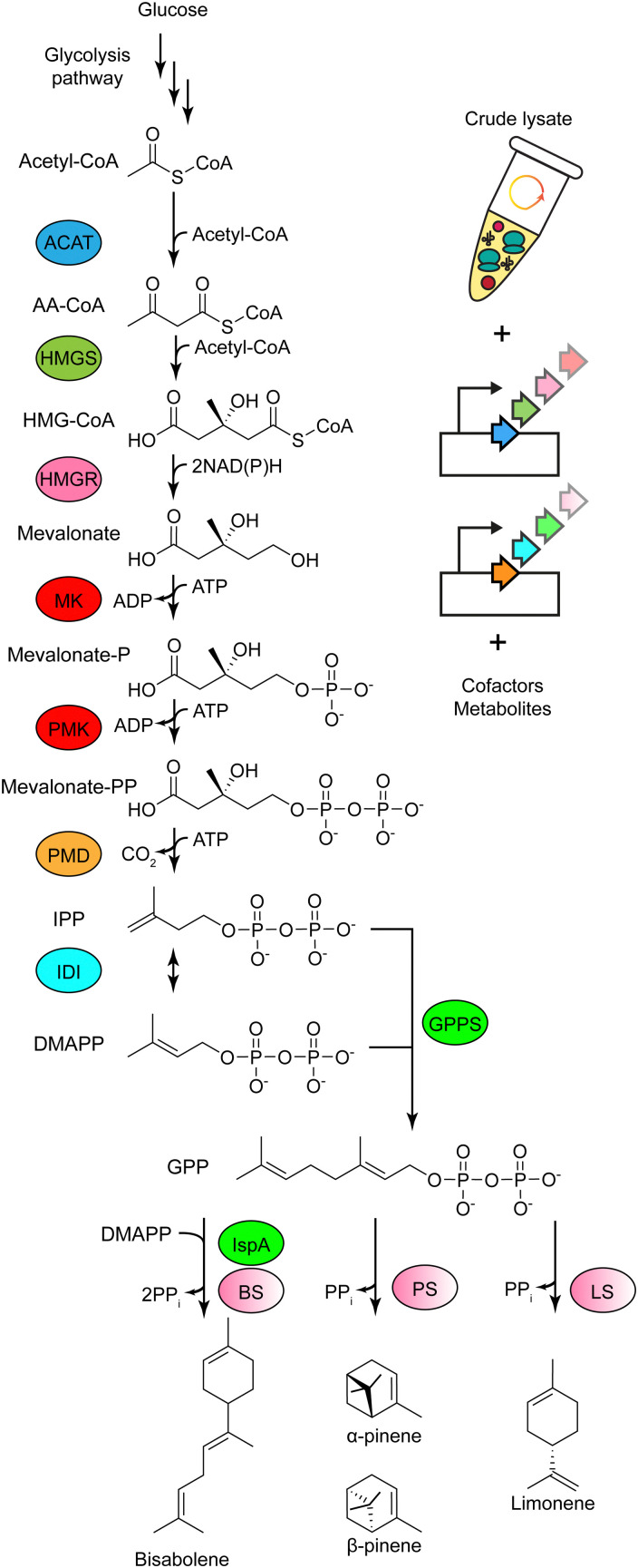
Cell-free biosynthesis of limonene, α/β-pinene and bisabolene. The authors used cell-free protein synthesis for rapid testing of different enzyme combinations to optimise and modularise the pathway.^142^ Abbreviations: AA-CoA = acetoacetyl-CoA, IPP = isopentylpyrophosphate, DMAPP = dimethylallyl pyrophosphate, GPP = geranyl pyrophosphate.

To date, the only terpene metabolites generated in a CFB system are limonene, pinene, and bisabolene,^145,146^ which built on prior efforts using an enzyme-based *in vitro* system for limonene generation.^357^ Importantly, this was the first crude extract-based system that had >5 enriched enzymes, encompassing 17 biosynthetic steps in total (Fig. 10). Briefly, a mevalonate system was reconstituted in *E. coli* BL21(DE3) star-based lysate and a set of six enzymes known to convert mevalonate to limonene was leveraged. For the glucose-to-limonene pathway, multiple modules of metabolism were strung together: glycolysis, an acetyl-CoA-to-mevalonate module, and the mevalonate to limonene module. This overall scheme was hindered because native *E. coli* farnesyl diphosphate synthase (IspA) was active within the lysate and diverted flux away from the intermediate geranyl pyrophosphate to farnesyl pyrophosphate and farnesol. With this knowledge, alteration of the cofactor pool enabled production of up to 3.8 mg L^−1^ h^−1^. Importantly, this biosynthetic pathway encompassed 20 biosynthetic steps, which showed the utility of CFE for much more complex metabolic networks.^145,146^

After initial efforts established that generating limonene in CFE was feasible, a cell-free approach called *in vitro* Prototyping and Rapid Optimisation of Biosynthetic Enzymes (or iPROBE) was developed. When using iPROBE, many pathway combinations can be rapidly built and optimised *via* separation into discrete reactions that are then mixed to assemble distinct biosynthetic pathways. The limonene pathway with nine biochemical steps served as an excellent test case to prototype. 150 unique sets of enzymes (particularly distinct homologs of enzymes) in 580 pathway conditions were screened using iPROBE to prototype the best metabolic pathway. This included distinct homologs of enzymes used to design the pathway and fine tuning of the cofactors, which turned out to be a critical parameter. This approach resulted in an increase in limonene production of 25-fold (from ∼23 to 610 mg L^−1^). To show the modularity and broader applicability of this approach, the terpene synthase enzymes were replaced and just the isoprenoid module was kept, allowing for the generation of the terpenes pinene and bisabolene.^145^

#### 4.4.2 Prospects for CFE of terpene biosynthetic pathways

Prior to using CFE to generate terpenes, purified enzyme systems showed that the level of metabolic complexity required for terpene biosynthesis could be achieved outside the cell. First, an investigation underwent substantial engineering efforts to generate limonene (12.5 g L^−1^) and pinene (14.9 g L^−1^), two chemicals responsible for important fragrances that give the aromas of citrus and pine, respectively. These levels are far higher than was observed in living systems. To do so, the enzymes breaking down glucose from glycolysis were reconstructed, as was a mevalonate pathway.^357^ A second study developed a similar platform for development of complex plant pathways *via* producing nepetalactol, an important precursor for strictosidine, a precursor to over 3000 members of the monoterpene alkaloid family. The monoterpene alkaloid family is responsible for several important natural products including strictosidine, a universal biosynthetic precursor, vinblastine an important anticancer agent, and ibogaine, an anti-addictive. Efforts to generate *cis*–*trans* neptalactol used a complex network of terpene synthases coupled to an extensive NADPH cofactor recycling pathway in a one pot synthesis.^358^ Such *in vitro* reconstitutions with enzymes set the stage for developing CFE approaches and illustrated the need for sophisticated cofactor recycling strategies.

## 5. CFE for antimicrobials and resistance

AMR is a pressing global challenge^359^ causing approximately 700 000 annual global deaths, and is predicted to rise to 10 million by 2050.^360^ Importantly, no new chemical classes of antibiotics have been approved for clinical treatment of Gram-negative bacteria since the synthetic fluoroquinolones developed during the 1970s.^361^ While there are several economic and scientific challenges to antibiotic discovery,^6,362^ there is a renewed desire to find novel chemical scaffolds and targets, as well as the use of non-standard antibiotics (*i.e.*, peptides, phages, nanomaterials).

In considering the challenge of AMR, antibiotics are one of the most significant discoveries of the 20th century, and a highly successful form of chemotherapy in terms of lives saved.^362–364^ While less studied, we will discuss the application of CFE for the initial drug screening process, and its potential for structure–activity relationship (SAR), antibiotic mechanism of action (MOA) and resistance studies.

### 5.1 Traditional antimicrobial screening

Antimicrobial discovery typically requires phenotypic whole cell bioactivity assays, selecting for compounds that either target the cell envelope or are favourably transported inside the cells to bind intracellular targets. Therefore, primary screening typically obtains low hit rates of discovery and is reliant on growth-inhibition (*e.g.*, agar diffusion, broth dilution) assays. The development of bioactive leads specifically for Gram-negative bacteria is a priority, including extended-spectrum beta-lactamases (ESBLs) and carbapenemase-resistant strains.^365,366^ Gram-negative bacteria are particularly insidious since they are protected from many antibiotics due to cell envelope barriers. This includes an inner membrane, peptidoglycan and outer membrane, as well as extracellular decorations, including sugar capsules (*e.g.*, *Klebsiella pneumoniae*) and lipids/polysaccharides. Together these components form a permeability barrier restricting access of antibiotics across the inner and outer membrane.^367^ Because of the differences in cell envelope physiology, bioactive compounds are easier to obtain for most Gram-positive bacteria. For antibiotic screening, most studies have prioritised bioactive compounds that permeate the cell envelope or target the cell membrane (*e.g.*, pore forming). However, previous screening of synthetic compound libraries suggest that finding compounds with structural properties that satisfy both bioactivity and cell permeability is challenging.^368^ In contrast, while antimicrobial natural products typically do not obey Lipinski's rule of five for drug-like properties,^4^ they have evolved to exploit specific transporters, such as TolC-BtuB^369^ and OmpA,^370^ to penetrate the cell envelope defences and engage their target(s).

The Nobel laureate Frances Arnold notably said, “you get what you screen for”.^371^ Systematic industrial antibiotic screening used growth inhibition, which led to the discovery of many important antibiotics from natural product libraries^5,372^ often containing complex mixtures of metabolites, and of variable concentration, quality, and chemical stability. However, considering that transport is limiting for many small molecules, whole cell screening only selects for the most abundant bioactive molecules or those with potent and favourable import into cells. While this is a drawback, cell-based growth inhibition screening assays remain the gold standard due to their success.^373^ In contrast to natural products, HTS screens of synthetic small molecule libraries has had limited success, in part due to non-specific activity of pan-assay interference compounds.^374^ In addition, with the rise of genomics technologies, antimicrobial discovery has also attempted target-based screening assays as a direct method for MOA elucidation. This approach investigates effects on a single purified protein or complex target, although there have been limited leads despite significant investment, largely due to poor translation of target binding into cellular potency.^368^ From 1995 to 2001, GSK evaluated over 300 target genes and 70 target-based HTS campaigns against their collection of 260 000–530 000 compounds, which resulted in five hits-to-leads.^375^ Although target-based assays are less successful than whole cell primary screening platforms for antimicrobial discovery, MOA studies such as topoisomerase inhibitor development have shown promise.^376,377^ Alternatively, screening against targets from pathogenic bacteria, instead of model organisms, increases the potential to identify narrow spectrum leads.^378,379^

### 5.2 Antibacterial mechanisms of action

Before considering the application of CFE for antimicrobial screening, we will briefly discuss four major targets in bacterial cells that antibacterial compounds act on, ultimately leading to cell death (bactericidal) or growth inhibition (bacteriostatic). The primary targets include (1) protein synthesis, (2) nucleic acid synthesis, (3) primary metabolism, and (4) cell wall synthesis and/or cell membrane disruption. Within the context of this review, protein synthesis is the major target, however, inhibitors have been demonstrated for DNA gyrase^165^ and energy regeneration^46,380^ targets. Several antibiotic classes target protein synthesis by binding to ribosomes at different locations. Aminoglycosides and tetracyclines interact with the 16S rRNA of the 30S subunit near the A-site, causing premature termination of mRNA translation.^381,382^ Kasugamycin, derived from *Streptomyces kasugaensis*, is an aminoglycoside that acts as mRNA mimic disrupting mRNA–tRNA codon-anticodon interaction.^383^ Macrolides, chloramphenicol derived from *Streptomyces venezuelae*, and synthetic oxazolidinones bind to the 23S rRNA of the 50S subunit at the polypeptide exit tunnel. Specifically, bulky macrolides bind the nascent peptide exit tunnel resulting in peptidyl-tRNA drop off and translation abortion while the other two have a π-stacking interaction that bind at the A-site of the peptidyl transferase centre and prevent binding of incoming charged-tRNA.^384,385^ The third major type of antibiotic targets includes nucleic acid synthesis and DNA repair mechanisms. Rifamycin sourced from *Amycolatopsis mediterranei*, and its semi-synthetic derivatives, including rifampicin, inhibit RNA synthesis by targeting DNA-dependent RNA polymerase at the DNA/RNA channel of the RNA polymerase β-subunit.^386^ This interaction blocks further RNA elongation. Quinolones (synthetic) target DNA gyrase and topoisomerase IV, which are involved in relieving torsional stress during DNA replication and supercoiling of bacterial chromosome.^387,388^ Alterations in supercoiling control leads to the formation of cleaved complexes (drug–enzyme–DNA), chromosomal fragmentation and ultimately cell death.^389^

### 5.3 CFE-based antimicrobial screening

**Fig. 11:**
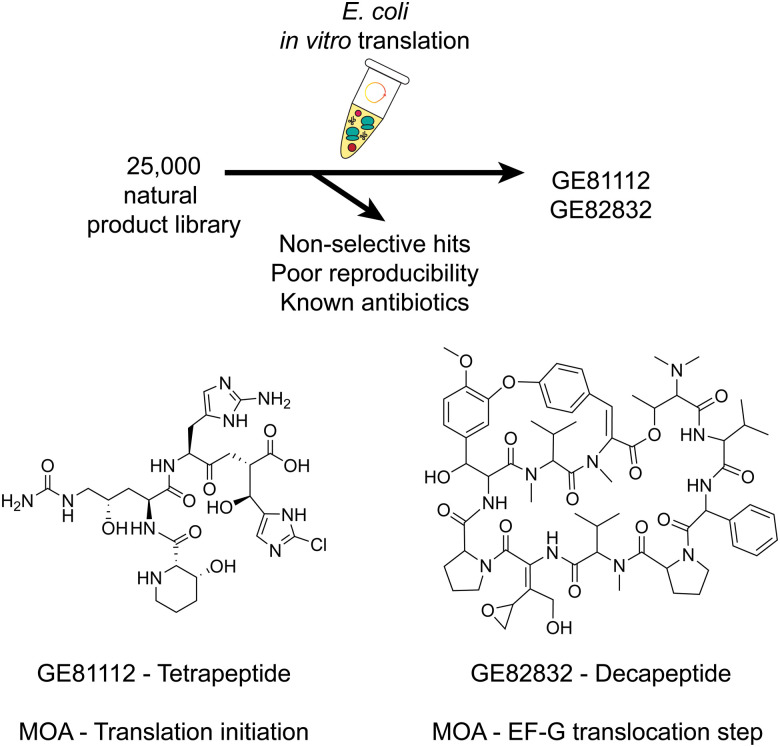
Outline of HTS of a 25 000 member natural product library using an *E. coli* IVT assay to discover GE81112 and GE82832, the former, a first in class antimicrobial disrupting the formation of the pre-initiation translation complex.^21^ The stereochemistry of GE82832 is not shown, as this was not reported.

In contrast to cell-based screening, CFE provides an alternative approach to antimicrobial discovery, with advantages and disadvantages (Table 5). First, a cell-free extract provides a low cost (<$0.10 per drug) screening assay for detecting antimicrobial compounds from natural product and synthetic sources. For comparison, most HTS assays cost $0.10–0.50 per drug for single target enzyme assays, or $1–10 for whole cell targets.^390^ Historically, IVT assays have been used to validate the MOAs of antibiotics and toxins, using *E. coli*, *Bacillus* sp. and cyanobacteria extracts.^151,391–396^ For example, a dipeptide antibiotic TAN1057 A/B (GS7128), derived from the soil bacterium *Flexibacter*, was discovered by a *E. coli* IVT system and determined to disrupt the peptidyl transferase reaction.^395^ Interestingly, GS7128 displayed whole cell activity, possibly through import by a dipeptide transporter. In addition, gene reporter systems have been developed for antibiotic interference such as translation initiation.^21^ Therefore, broadly, IVT assays have provided a tool for characterising novel antimicrobial activity.^397–400^ In the early 2000s, a dual *E. coli* and *S. cerevisiae* IVT assay based on a single mRNA transcript^21^ was developed to verify target specificity and HTS of 25 000 microbial natural product extracts (Fig. 11). This led to the discovery of GE81112, a tetrapeptide inhibitor of prokaryotic translation initiation and first in class hit.^401^ GE81112 was later established to be a non-ribosomal peptide derived from a 61 kb BGC from *Streptomyces* sp. L-49973.^402^ Besides GE81112, the cyclic peptide GE82832 was also discovered through IVT and shown to inhibit translocation by binding to the 30S subunit.^403^ However, despite demonstrating novelty in terms of MOA and chemical structures, IVT screening encountered issues such as a high hit rate, which is unfavourable for industrial screening. In addition, the assay enriched for novel compounds that showed high activity *in vitro* but unfortunately had limited activity in whole cell assays. However, while GE81112 had poor transport into bacterial cells, structural derivatives were later developed with improved membrane permeability.^42,404–407^ This evidence supports that CFE approaches have the potential to deliver hit novelty in terms of MOA, whereas activity in cells can later be achieved through rational drug design to reach a lead. Alternatively, by focusing on natural product extracts displaying both *in vitro* and whole cell activity to reduce the hit rate, additional work led to the discovery of orthoformimycin from *Streptomyces* sp. ID107558. This compound inhibited EF-G dependent movement of the mRNA on the ribosome and Pi release from EF-G without affecting EF-G dependent aminoacyl-tRNA translocation.^21,408^ Employing reporter systems in CFE also aids the detection of novel gene expression inhibitors.^409^

**Table 5:** Comparison of antibiotic screening primary assays. Whole-cell assays are broth dilution or disc-diffusion formats with minimum inhibitory concentrations. Target-based assays measure antibiotic action using enzymatic reaction readouts or binding. CFE measures antimicrobial inhibition of mRNA/protein synthesis

Assay	Whole-cell assay	Target-based assay	CFE
Screening format	Growth inhibition	Effect on pre-determined target	Inhibition of mRNA and protein synthesis
Advantages	Standardised method	HTS	HTS
	Translation into clinic	Mechanism of action	Mechanism of action
			Structure–activity relationship
	Physiological effect	Structure–activity relationship	Mimics native cytoplasmic environment
			Multiple intracellular targets
Limitations	Mechanism of action unknown	Characterised target	Hits may not be cell permeable
	Scalability to HTS	Purified target required	Low permeability – limited to cellular activity
	Permeability barrier – false negatives	Low hit rate	

The ribosome and the core transcription and translation machinery are both highly conserved within, and evolutionarily divergent between, prokaryotes and eukaryotes, which enables selectivity in terms of antibiotic drug design. However, there are substantial differences between individual microbial cell types across all levels of genetics, biochemistry and physiology that could be exploited for the development of narrow-spectrum antimicrobials. While antimicrobial screening has previously focused on broad spectrum approaches (*i.e.*, Gram-negative and Gram-positive bioactivity), there is more scope for species-specific targeted development of antimicrobials as measure to minimise the risk of AMR.^361,410^ For this purpose, some cell-free studies have developed species-specific CFE tools. For example, a *Staphylococcus aureus* CFE assay was developed to evaluate transcription–translation inhibitors from a cytoplasmic extract and purified ribosomes.^411^ Then, a HTS platform was developed for a clinically relevant *Streptococcus pneumoniae* strain and used to screen 220 000 compounds using a dual translation-activity assay with luciferase as reporter.^152^ Identified hits were screened in a panel of species-specific CFE assays to evaluate specificity. A limitation of this study was the high rate of off-target inhibition of the luciferase enzyme, leading to false-positives. More recently, *Klebsiella pneumoniae* CFE was developed from laboratory and clinical strains and used for the study of AMR genetic determinants, as well as for targeted antimicrobial characterisation to explore differences between *K. pneumoniae* and an *E. coli* CFE model.^165^ This showed that the minimum concentration of antibiotic to inhibit 50% of *K. pneumoniae* cells (MIC_50_) ranged between one to two an orders of magnitude higher (with the exception of chloramphenicol and tetracycline) than the equivalent 50% inhibitory concentration (IC_50_) value determined with *K. pneumoniae* CFE for most antibiotics tested.^165^ This was insightful, since past industrial-led antimicrobial screening with whole cell assays was typically performed with complex natural product extracts and fractionated libraries, derived from laboratory grown microbial isolates, and diversified through temporal and geographic sampling from biologically diverse regions.^21,412,413^ This is important because natural products are typically only produced at low concentrations by laboratory grown microbes. Potentially, CFE could offer an opportunity to detect low abundance bioactive compounds from complex natural product libraries otherwise missed by whole cell assays.^21,42,152,401^

### 5.4 CFE as a tool to study antibiotic resistance

AMR arises through adaptive chromosomal or plasmid mutations in response to antibiotic-induced cell stress, or through acquisition of AMR genes (ARGs) from the environment.^414^ Resistance mechanisms are grouped into six categories: (1) reduced permeability, (2) increased efflux of antibiotics from the periplasm and cytoplasm, (3) modification of antibiotic target, (4) target mimicry, where an evolved ortholog bypasses an inhibited metabolic step, (5) antibiotic inactivation by modification, sequestration, or degradation, or (6) biofilm formation.^414^

For profiling of antibiotic resistance, there are two predominant approaches: genotyping and phenotyping. Genotyping uses whole-genome next-generation sequencing (NGS) and bioinformatics tools, such as RESfinder,^415,416^ to detect known genetic determinants associated with AMR (*i.e.*, resistance genes, single-nucleotide polymorphisms in AMR determinants). Uniquely, NGS can study single strains and mixed populations. However, both genotyping and bioinformatics analysis still requires laboratory-based phenotypic experiments, especially for emerging resistance determinants. Phenotyping requires cell culture, broth dilution, time kill, and disc diffusion assays, following the recommended standards from the U.S. Food and Drug Administration (FDA), World Health Organization, National Committee for Clinical Laboratory Standards and EUCAST. Critically, the MIC_50/90_ value is a complex measure of overall antibiotic activity, a combination of antibiotic import, efflux, metabolism, selectivity, and potency, all overlaid by mutations associated with the resistome.^417^ Additionally, mutations associated with a specific AMR phenotype can confer collateral resistance or sensitivity to structurally or functionally unrelated antibiotics.^418–420^ Similarities occur in cancer drug resistance.^421^

As an extra tool for phenotypic analysis of AMR, several studies have reported the use of IVT and CFE tools for mechanistic studies of resistance, using either crude extracts or purified ribosomes. For example, purified ribosomes from a mutant *E. coli* strain containing single or double 16S rRNA mutations (U1060A and U1052G) in the head domain of 30S ribosomal subunit were investigated.^422^ U1052 16S rRNA mutants increased resistance to negamycin and susceptibility to tetracycline resulting from an unexpected tetracycline-binding site.^422^ Ribosomal resistance from antibiotic-producing *Streptomyces* strains in CFE also provide insights into the protective mechanism against pactamycin (a ribosome inhibitor) specific to this host.^423^ Additionally, a small pentapeptide encoded within the *E. coli* 23S rRNA, if overexpressed, confers resistance to erythromycin.^424^ However, while the RNA encoding the pentapeptide conferred erythromycin resistance in an *E. coli* translation system, the corresponding synthetic peptide did not, suggesting the peptide has a short half-life and requires continuous expression.^424^ In related studies where AMR and drug discovery were not the focus, the use of CFE to study ribosomal mutants was established. Seven isolated ribosomal mutants from streptomycin-resistant *E. coli* BL21 were used to quantify CFE of chloramphenicol acetyltransferase.^425^ Most mutants showed reduced ribosomal activity and more interestingly increased translation accuracy measured by mis-incorporation of Leu in a poly(U) encoded poly-Phe sequence. A yeast CFE carrying ribosomal mutations in the S28 and S3 proteins (S12, S5 homologs in *E. coli*) was used to contrast translational accuracy and sensitivity/resistance to paromomycin.^426^ In addition, clindamycin-resistance was used as a selection approach to validate an *in vitro* ribosome synthesis and evolution *via* ribosome display technology.^427^ A fungal IVT system resistant to the translational inhibitor trichothecin^428^ was also investigated. Collectively, these studies reveal the potential of CFE to reconstitute mutant ribosomes and study the differential effects on antibiotic resistance, ribosomal processivity and translational accuracy.

**Fig. 12:**
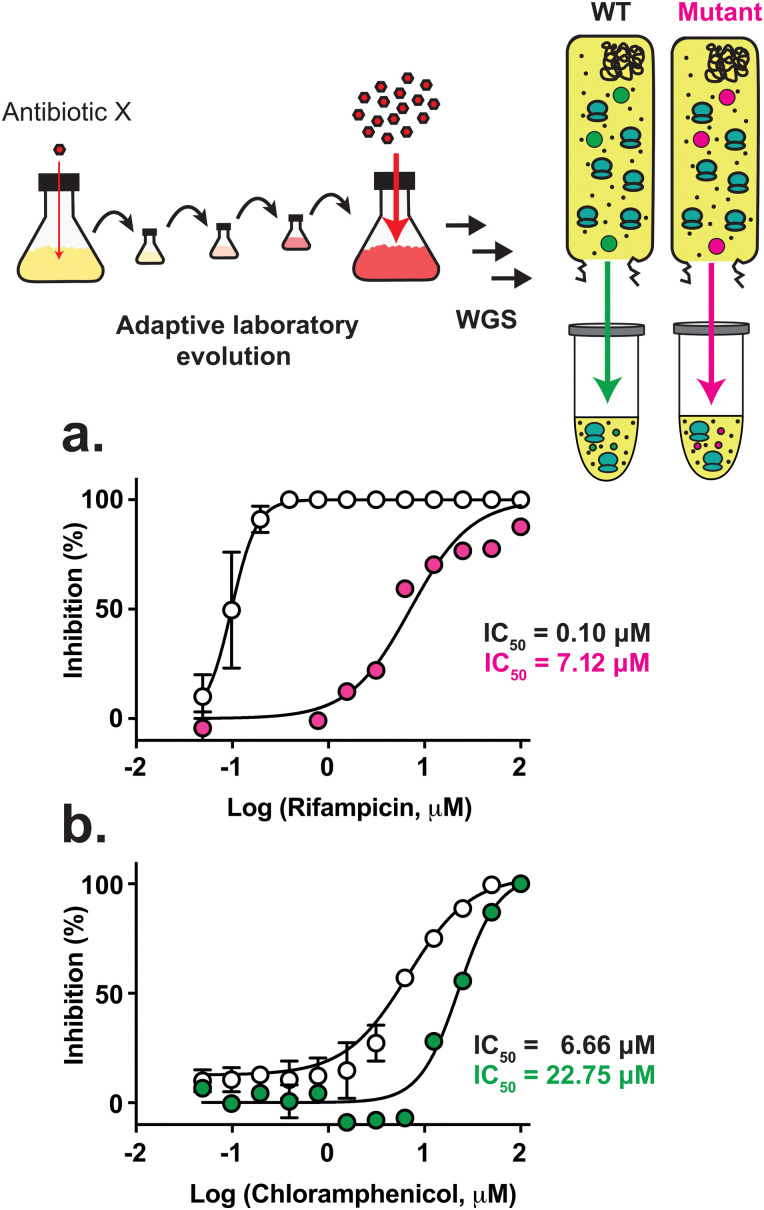
The application of a *K. pneumoniae* CFE tool to study antibiotic resistance associated mutations and their effects on protein synthesis for (a) rifampicin resistance, and (b) chloramphenicol resistance. Figure reproduced and adapted with permission.^165^

CFE is a viable tool to profile ARGs. However, except for chloramphenicol acetyltransferase, which historically was used as a gene expression reporter with CFE,^429^ only a handful of studies, mostly during the 1980s, have used IVT/CFE to study ARGs. For example, both *Streptomyces* and *E. coli* CFE were used to express and identify resistance gene products against novobiocin,^430^ hygromycin B,^431^ and thiostrepton.^285^ More recently, a *K. pneumoniae* CFE tool was combined with adaptive laboratory evolution to study chloramphenicol (50S ribosome), valnemulin (50S ribosome), and rifampicin (RNA polymerase) resistance.^165^ Genomic sequencing of a selection of these mutants was performed to determine the genotype, followed by phenotypic characterisation of the extracts derived from these strains, through measuring protein synthesis and resistance to these set gene expression inhibitors. Specifically, an RNA polymeraseβ-subunit (*rpoB*) H526L mutant displayed 40-fold increased resistance to rifampicin, while moderate resistance was observed for chloramphenicol (Fig. 12). Outside of academia, Biobits® has made a cell-free educational kit for expressing an aminoglycoside kinase for inactivating the translational inhibitor kanamycin.^432^

### 5.5 Prospects for CFE for antimicrobials and resistance

Overall, CFE technologies can serve a range of uses within antimicrobial discovery, and the study of antimicrobial resistance. Key advantages include the speed, cost, and potential for automation, while natural products are notoriously produced at limited quantities, whereas cell-free bioassays are shown to have orders of magnitude higher sensitivity than conventional whole cell antimicrobial assays. We suggest CFE provides a rapid and flexible research tool to support the characterisation of new ARGs and resistance profiles that will become more prevalent with the rise of AMR and the use of new antibiotics. There is also potential to employ CFE to mimic the biochemistry of a cell and study structure–activity relationship studies for antimicrobial development. In addition, there is evidence CFE is beneficial for identifying novel modes of action, such as peptide-based antimicrobials.

## 6. Beyond the lab: cell-free technology in industry

**Fig. 13:**
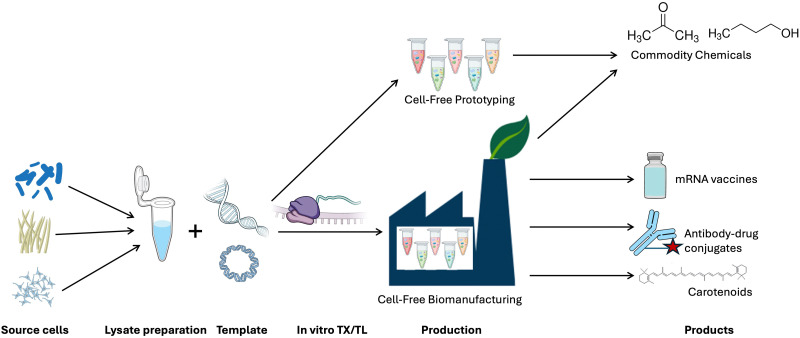
Overview of industrial strategies to apply CFE as a prototyping and production platform.

Finally, we shall consider the wider use of CFE and CFB within industry (Fig. 13). Cell-free (both CFE and extract) approaches are attractive for industrial biomanufacturing because of separation of biomass growth (for lysate production) and product formation (performing biosynthetic reactions using the lysate), increased biosynthesis rates due to the abolishment of the need for transport of substrates and products across membranes and/or cell walls, and substrate and product tolerance. Owing to technology advances and scale-up capabilities, cell-free is emerging as an attractive and alternative platform for development and commercialisation of next-generation pharmaceuticals, cosmetic and food ingredients and specialty and commodity industrial chemicals. A recent analysis showed that cell-free patent filings increased five-fold over the last decade (compared to just a 1.5-fold increase in peer-reviewed publications) with an increase in company filed patent applications by seven-fold between 2015 and 2020.^2^ Here we briefly describe some companies and applications of cell-free biomanufacturing they are employing and note whilst these are largely not-focused on natural products *per se*, are illustrative of commercial applications that could be employed for natural products.

LanzaTech, a company that uses gas fermentation with *Clostridium autoethanogenum* for the production of commodity chemicals, has developed a cell-free prototyping platform that enabled them to identify enzymes causing unwanted side reactions which they then eliminated from the genome, resulting in increased product titers of acetone and isopropanol.^433^ This platform, termed iPROBE (discussed specifically in Section 4.4.1) allows for the rapid prototyping of biosynthetic pathways, combinatorial pathway design and testing, and pathway optimisation.^434^ This two-step system is initiated with CFE synthesis of desired enzymes and is followed by the reaction phase upon addition of substrates and cofactors. They also achieved increased production of 3-hydroxybutyrate, 2,3-butanediol and butanol, showing the versatility and usefulness of this platform.^434^

Other companies are using cell-free technology to produce vaccines and therapeutics. Nature's Toolbox Inc.^435^ has developed a continuous flow CFE using hollow fibre bioreactors for production of RNA molecules, especially those mRNA for therapeutics, that provides flexibility and accelerated production compared with traditional cell-based or synthesis systems. Such an approach could be highly valuable when rapid responses are required (*e.g.* such as in pandemics). They also offer a cell-free protein expression platform. Vaxcyte^436^ is using their CFE platform, licensed from Sutro Biopharma,^437^ to engineer, optimise and manufacture vaccines, focusing on vaccines for invasive pneumococcal disease, Group A Strep, periodontitis and Shigella. They couple CFE with conjugate chemistry to add functionality. Sutro Biopharma has developed CFE that incorporate non-natural amino acids (nnAAs) using orthogonal translation systems consisting of non-native tRNAs and aminoacyl-tRNA synthetases that lead to single-species antibody–drug conjugates for antitumor applications. Ginkgo Bioworks recently entered into an agreement with DARPA to deliver therapeutic proteins such as antibodies, cytokines and clotting factors using cell-free methods in conjunction with their biofoundry. Resilience acquired Swiftscale Biologics^438^ CFE platform and now uses that for production of various biologics.

Several companies have developed CFB catalytic processes for products across multiple applications. FabricNano^439^ uses enzyme immobilisation to conduct biocatalysis for a range of applications. They have built and offer a platform for predictive immobilisation (Predictive Immobilisation Plates) which allows for optimisation of enzyme and immobilisation methods in a combinatorial manner that reduces the need for mass experimentation. They have partnered with several companies. EnginZyme has developed a patented process for pseudouridine biosynthesis using immobilised enzymes, that leads to a purer version of pseudouridine compared to conventional synthesis approaches. *N*1-Methylpseudouridine-5′-triphosphate, which is derived from pseudouridine, helps stabilise and reduce the immunogenicity of mRNA vaccines, such as that for COVID-19.

Besides the animal cell and microbial CFE platforms described above, plant CFE have also been developed and commercialised. Most notably is the development of tobacco BY-2 cell suspension cultures that have achieved protein yields of 3 mg mL^−1^ in a coupled CFE reaction and have been used for the production of lycopene, indigoidine, and betalains.^440^ This system has been commercialised by LenioBio GmbH as ALiCE® (Almost Living Cell-free extract) and further been shown to produce virus-like particles, membrane receptors and a monoclonal antibody.^441^

The advent and application of artificial intelligence has been applied to cell-free technology. While various academic efforts that combine automation, artificial intelligence and cell-free technology, the commercial sector is also seeing these combined technologies being employed. Debut^442^ has a scalable cell-free platform that spans discovery to commercialisation and who is focusing on products for the personal care industry. Tierra Biosciences^443^ is combining cell-free protein expression with AI for on-demand and custom protein synthesis.

Broader adoption of cell-free technology in industrial biomanufacturing has been hampered by the costs and scale of lysate preparation, cofactor provision, and the ability to operate in a continuous mode. It has been calculated that it costs approximately $5000 per L for a standard cell-free reaction formulation with most of the cost coming from the phosphorylated energy substrate, phosphoenolpyruvate (a detailed breakdown of these costs can be found in the Supporting reference of a comprehensive review by Silverman *et al.*).^1^ As described above, the commercial applications are predominately in the therapeutic space, where the economics can sustain these higher costs, and where scales are diminished compared to commodity chemicals. With further optimisation and development work, alternative approaches to mitigate such economic and scale issues could be possible which will open new avenues for cell-free biomanufacturing.

## 7. Future outlook

The ability to use CFE and cell-extract based technologies for a variety of applications has exploded in recent years. However, applying the wealth of options to generate complex proteins, pathways, and ultimately small molecule metabolites has only recently been directed towards applications relevant to the expression of biosynthetic gene clusters and their corresponding natural products. To date, preliminary efforts to generate peptide, polyketide, and terpene natural products have been performed both in lysate-based and PURExpress® systems. These initial studies can be directed in multiple ways to further natural products research. Generating uncharacterised natural products in CFE can be used to access compounds that are not readily biosynthesised in their producing organisms under laboratory conditions and can produce molecules that present toxic effects when overexpressed in heterologous hosts. CFE can also be used to rapidly evaluate modes of activity, such as antibiotic resistance.

Prototyping design for bioengineering applications remains a huge unrealised application of CFE. Due to the complexity of these metabolic pathways, there are numerous avenues for optimisation both of precursor flux and of pathway design. For the so-called “megasynthase” enzymes (PKS and NRPS enzymes), using this iterative “design-build-test-learn” cycle has promise for designing chimeric assembly lines.^444–446^ Efforts to generate soluble, full-length proteins present a major step towards this application.^266^

For RiPPs, as the precursor is a proteinogenic amino acid, there are many opportunities for pathway elucidation and engineering.^167,172,447^ One of several challenges in CFE for RiPPs natural products is establishing appropriate cofactor recycling for redox sensitive enzymes, such as radical SAM enzymes. Recent progress in strategies for complex cofactor recycling, such as efforts to generate iron sulphur clusters under aerobic conditions,^448^ may further expand the application of CFE in RiPPs. Terpenes have been shown to be highly modular for bioengineering applications *in vivo*^449^ as evidenced by efforts to produce complex terpene natural products (a key example being artemisinin^450^) in tractable heterologous hosts. Metabolic engineering approaches such as iPROBE demonstrates that similar engineering approaches can be employed in cell-free environments.^145^ Furthermore, once generated, CFE can be used to rapidly evaluate bioactivity of these compounds.

In summary, advances in technology continue to push the horizons of what is achievable in CFE. Whether it is democritising the PURE® system,^117^ improving co-factor recycling, or adapting novel lysates to cell-free applications with natural products, these advances highlight continued development of these robust platforms. Ultimately, this will allow biomanufacturing to extend to a broader range of metabolic pathways and expand the synthetic biology of natural products.

## Author contributions

AJR, TTS, KC, DAM, NJM, SJM, and CBM wrote the manuscript.

## Data availability

No primary research results, software or code have been included and no new data were generated or analysed as part of this review.

## Conflicts of interest

There are no conflicts to declare.
